# Possible Genetic Risks from Heat-Damaged DNA in Food

**DOI:** 10.1021/acscentsci.2c01247

**Published:** 2023-06-01

**Authors:** Yong Woong Jun, Melis Kant, Erdem Coskun, Takamitsu A. Kato, Pawel Jaruga, Elizabeth Palafox, Miral Dizdaroglu, Eric T. Kool

**Affiliations:** ^†^Department of Chemistry, ^⊥^Sarafan ChEM-H, and ^#^Stanford Cancer InstituteStanford University, Stanford, California 94305, United States; ‡ Biomolecular Measurement Division, National Institute of Standards and Technology, Gaithersburg, Maryland 20899, United States; § Institute for Bioscience & Biotechnology Research, University of Maryland, Rockville, Maryland 20850, United States; ∥ Department of Environmental & Radiological Health Sciences, Colorado State University, Fort Collins, Colorado 80523, United States

## Abstract

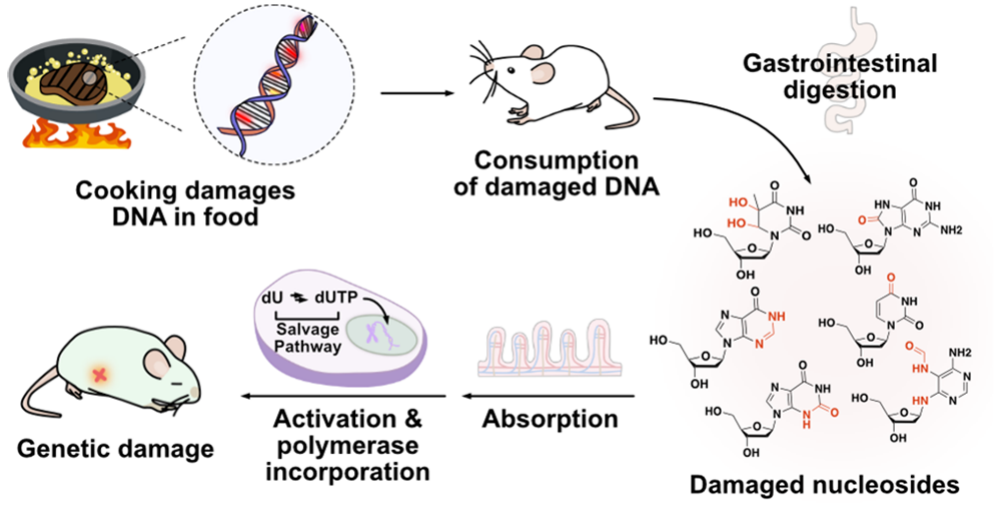

The consumption of foods prepared at high temperatures has been associated with numerous health risks. To date, the chief identified source of risk has been small molecules produced in trace levels by cooking and reacting with healthy DNA upon consumption. Here, we considered whether the DNA in food itself also presents a hazard. We hypothesize that high-temperature cooking may cause significant damage to the DNA in food, and this damage might find its way into cellular DNA by metabolic salvage. We tested cooked and raw foods and found high levels of hydrolytic and oxidative damage to all four DNA bases upon cooking. Exposing cultured cells to damaged 2′-deoxynucleosides (particularly pyrimidines) resulted in elevated DNA damage and repair responses in the cells. Feeding a deaminated 2′-deoxynucleoside (2′-deoxyuridine), and DNA containing it, to mice resulted in substantial uptake into intestinal genomic DNA and promoted double-strand chromosomal breaks there. The results suggest the possibility of a previously unrecognized pathway whereby high-temperature cooking may contribute to genetic risks.

## Abstract 2

Cooking food at high temperature is found to damage DNA in foods. Incubating damaged DNA components with cells, or feeding them to mice, results in damage to genomic DNA.

## Introduction

Cooking foods at high temperatures has been associated with numerous health risks.^1^ The consumption of red meat, which is frequently prepared at high temperature, is associated with colorectal and pancreatic cancer as well as metabolic syndromes such as type 2 diabetes and cardiovascular disease, and this consumption is also negatively associated with longevity.^2^ High-temperature cooking of certain vegetables for consumption is also associated with disease risk.^3^ Numerous mechanistic studies have implicated chemical changes in cooked food with damage caused to human DNA.^1,4^ This has led the Food and Drug Administration (FDA) to recommend reductions in the public consumption of red meat and of deep-fried foods in general.

**Figure 1:**
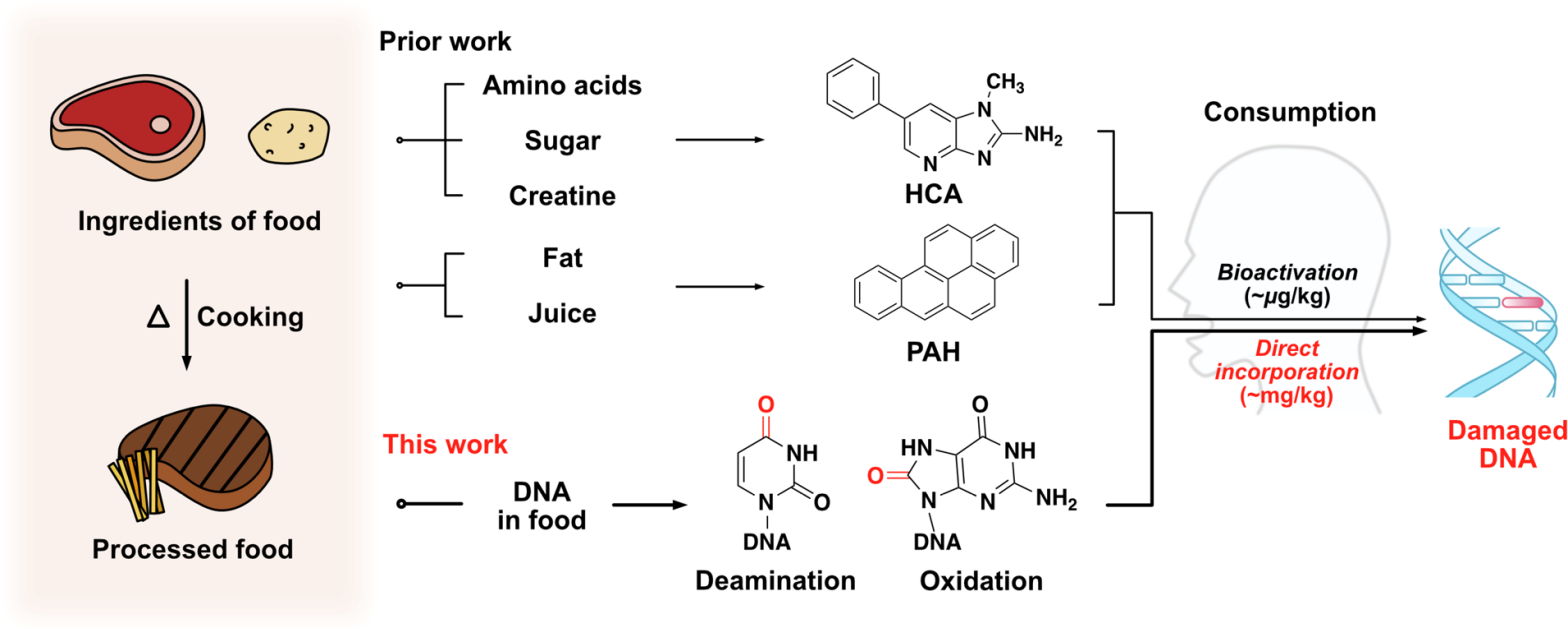
Prior studies have identified small-molecule metabolites (e.g., HCA and PAH) produced at trace levels during cooking that can alkylate human DNA after bioactivation. Our hypothesis describes a potentially more direct and previously undescribed route, whereby consumption of heat- and air-damaged DNA in foods results in direct incorporation of the damaged components into the DNA of mammalian tissue. Critical steps of this process are (i) heat-induced damage to food DNA; (ii) consumption and digestion of food DNA into 2′-deoxynucleotides; (iii) uptake of damaged 2′-deoxynucleosides into cells and activation via the salvage pathway; and (iv) polymerase incorporation into cellular DNA. This has the potential to lead to serious DNA lesions including mutations, abasic sites, and double-strand breaks.

Studies aimed at delineating possible mechanisms of these pathologic associations have focused on small-molecule metabolites that can react with DNA. For example, polycyclic aromatic hydrocarbons (PAHs) and heterocyclic amines (HCAs) are produced at trace levels during the cooking of food and then bioactivated upon consumption into reactive species that alkylate DNA, resulting in the accumulation of damage and mutations over years of exposure (Figure 1).^5^ Other reactive and potentially carcinogenic small molecules generated during high-temperature cooking include aldehydes, acrylamide, and *N*-nitroso compounds which can alkylate DNA bases.^1^ When such species react with DNA, this can result in mutations when replication specificity is altered by modified nucleobases and in genotoxicity and chromosomal rearrangements when strand breaks occur during repair.

### Food DNA Damage Hypothesis

Significantly, very little research attention has been paid to the effect of elevated cooking temperatures on the DNA in the food itself. DNA is one of three major classes of macromolecules in mammalian cells, accounting for 0.3% of cellular mass;^6^ this implies that the consumption of a 500 g steak results in the ingestion of >1 g of DNA (Table 1). Moreover, elevated temperatures have been shown to have adverse effects on DNA integrity in DNA samples *in vitro*.^7,8^ The lack of studies on the effects of DNA damage in food may be due in part to the perception that ingested DNA is not likely to be taken up in cells to influence cellular pathways.^9^ However, it has long been recognized that DNA, when fed orally to mammals, is rapidly fragmented and hydrolyzed, ultimately to 2′-deoxymononucleotides (chiefly, 5′-monophosphates) by nuclease enzymes present in pancreatic and intestinal juices.^9,10^

**Table 1:** DNA Content of Selected Animal and Plant Tissues^9,17^

	Food		DNA (g/kg of dry matter)
Meats	Beef	Liver	19.5 (18.9 to 20)
		Heart	5.3
		Pancreas	16.2 (14.4 to18)
	Pork	Liver	14.8 (14.4 to 18.1)
		Heart	6.9
		Pancreas	21.2 (18.8 to 23.6)
	Horse	Muscle	9.2
Plants	Wheat		0.6
	Lentil		0.8 (0.7 to 0.8)
	Broccoli	Fresh	5.1
	Cauliflower	Fresh	2.8
	Spinach	Frozen	2.6
	Potato	Fresh	1
	Onion	Fresh	0.7
	Avocado	Fresh	0.6

In addition, 2′-deoxynucleoside 5′-monophosphates are dephosphorylated by 5′-nucleotidase (intestinal phosphatase) activities in the cell membrane,^10^ and the resulting free nucleosides (at least the canonical cases) can be taken up into the intracellular environment and participate in nucleotide salvage pathways (Supporting Information Figure S1).^9^ Interestingly, although the cellular nucleotide salvage pathway has been well studied with regard to canonical nucleosides/nucleotides, very little is known about the capability of damaged 2′-deoxynucleosides to be taken up into cells and incorporated into DNA there.^11^ However, if damaged 2′-deoxynucleosides were indeed taken up in salvage pathways, then this might present a significant risk by the direct placement of damage in host DNA.

Taken together, these issues combine to present a potential mechanism whereby the ingestion of damaged DNA from cooked food might result in the incorporation of plant- or animal-derived damaged nucleosides into human DNA, resulting in genetic lesions and possible health risks. As a result, it could potentially be of significant health interest to determine to what degree high-temperature cooking can result in damage to the DNA in food sources and if damaged DNA can be digested into damaged nucleosides and indeed could have the capacity to enter human nucleotide salvage pathways and be incorporated into cellular DNA. We are aware of no previous studies of these issues.

Early studies of DNA stability *in vitro* have shown that elevated temperature (milder than that employed in many cooking procedures, Table S1) can accelerate the deamination of 2′-deoxycytidine (dC) in DNA, resulting in 2′-deoxyuridine (dU),^7^ and also promotes the oxidation of guanine, resulting in the formation of 8-oxo-2′-deoxyguanosine (8-oxo-dG) along with other modified deoxynucleosides.^8^ 2′-Deoxyuridine, if incorporated into DNA by polymerase enzymes, is a targeted substrate for base excision repair (BER),^12^ and high levels of dU in cellular DNA can result in elevated numbers of single-strand nicks and, if proximally localized, double-strand breaks (DSB), leading to genotoxicity and genomic rearrangements.^13^ Many damaged 2′-deoxynucleosides such as 8-oxo-dG in DNA are highly mutagenic when incorporated and also can be genotoxic both in mitochondrial and nuclear DNA when subject to DNA repair.^14^ Indeed, because damaged nucleotides (when generated directly in cells) are potentially harmful, nucleotide pool sanitation enzymes exist to prevent their misincorporation into DNA via inactivation of their 5′-triphosphate derivatives.^15^

We emphasize that this overall hypothesis cannot be proven in such an initial study. Indeed, studies of small-molecule agents such as PAH and HCA in cooked foods have proceeded over decades, and risks to humans are seen only in large population studies. Thus, our goal is to test the individual parts of the food DNA hypothesis, which may lead to insights into its feasibility. To examine these hypothesized issues, we addressed three chief questions regarding the potential connection of the cooking of food and DNA damage in human DNA: First, to what extent does cooking cause damage to DNA in food? Second, does cellular exposure to damaged 2′-deoxynucleosides evoke DNA damage repair responses or chromosomal damage? Third, to what degree are damaged DNAs digested and salvaged by cells and incorporated into cellular DNA?

## Results and Discussion

### Cooking Results in High Levels of Damage to DNA in Food

**Figure 2:**
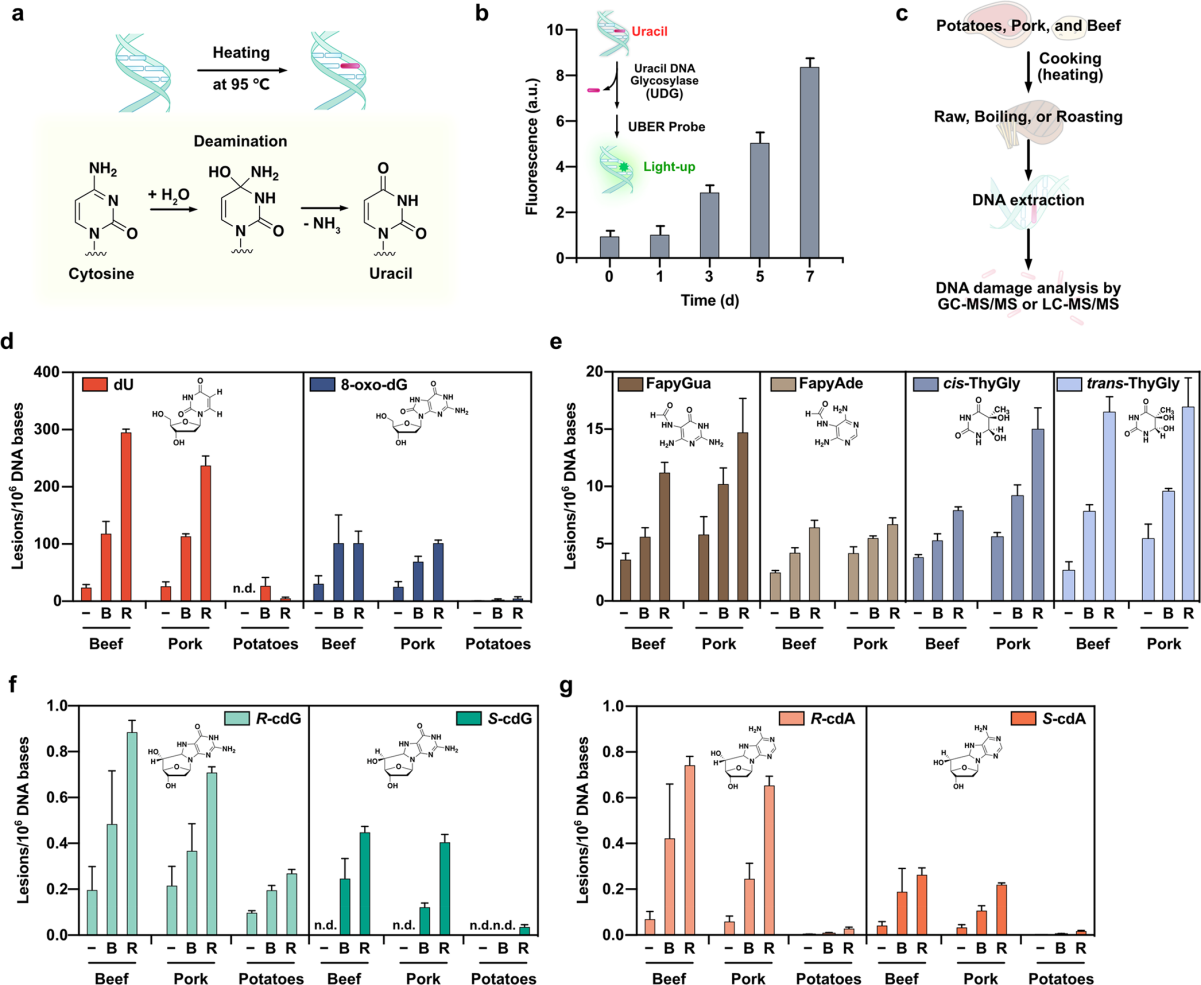
Measurements of specific forms of damage in DNA from food after heating and cooking reveal elevated levels of damage. (a) Illustration of deamination of cytosine affording uracil in DNA. (b) Uracil quantification assay in gDNA extracted from HeLa cells, employing UDG and a fluorescent probe for AP sites. (c) Procedure of DNA damage quantification with GC–MS/MS and LC–MS/MS in DNA extracted from food samples. (d–g) Levels of 10 types of DNA damage quantified with GC–MS/MS and LC–MS/MS in DNA extracted from raw (−) and cooked (B = boiled, R = roasted) food samples. n.d. = not determined. Cooked foods were boiled (100 °C, 20 min) or roasted (220 °C, 15 min) before DNA extraction. Uncertainties are standard deviations.

We tested the *in vitro* thermostability of genomic DNA (gDNA) extracted from HeLa cells, focusing on the deamination of cytosine, the most frequent form of heat-induced DNA damage *in vitro*.^7^ The extracted gDNA was subjected to extended heating (95 °C) to accelerate the deamination of cytosine to uracil in DNA (Figure 2a), and then the levels of uracil were measured with uracil-DNA glycosylase (UDG) and a fluorescence probe (UBER)^16^ specific to apyrimidinic/apurinic (AP) sites in DNA (Figure 2b). The results show that heating DNA at this elevated temperature markedly increased the level of uracil in DNA over time, as a result of the accelerated deamination reaction.

Cooking processes commonly involve temperatures much higher than 95 °C for minutes to hours (Supporting Information, Table S1), which suggests the possibility of significant DNA damage in food. Our initial observation of the elevated deamination of cytosine in extracted gDNA after heating prompted us to test the stability of DNA in food during cooking processes, focusing on multiple aspects: (i) To what extent is DNA in food damaged upon cooking and (ii) how does the type of food source and method of cooking affect damage? Ground beef (80% lean), ground pork (80% lean), and sliced potatoes were cooked via boiling for 20 min or roasting for 15 min in an oven (220 °C), and then DNA was isolated from the heat-processed foods as well as from uncooked controls (Supporting Information Figure S2). We employed gas chromatography/tandem mass spectrometry (GC–MS/MS) and liquid chromatography/tandem mass spectrometry (LC–MS/MS) to identify and quantify distinct chemical forms of DNA base damage in the raw and cooked foods. Structures of the DNA lesions measured are shown in Supporting Information Figure S3 and in Figure 2d–g.

The analysis showed that the levels of all 10 DNA lesions tested were significantly increased in the DNA extracted from heat-processed foods compared to those in the raw foods (Figure 2d–g,). FapyAde, FapyGua, *cis*-ThyGly, and *trans*-ThyGly could not be detected in DNA samples extracted from potatoes. For the meat sources, the higher temperature of cooking (roasting) generated greater amounts of DNA damage than the lower-temperature cooking procedure (boiling). In absolute terms, the two most frequent forms of damage were dU (10-fold increase after roasting) and 8-oxo-dG (3.5-fold increase after roasting). Relative to control levels (Supporting Information Figure S3), dU and 8,5′-cyclopurine-2′-deoxynucleosides (8-fold increase in *R*-cdA after roasting) were increased by the greatest factor. dU was found at levels of ∼300 bases per million nucleotides in meats after mild roasting (15 min) (Figure 2d). Given that heat-induced deamination producing dU in isolated DNA continues to proceed over extended times (Figure 2b),^18^ hours of roasting or smoking could potentially result in higher levels of damage, although this was not tested here. For dU in briefly roasted beef, the amounts found here correspond to milligram quantities in a serving of cooked meat, as much as 1000 times greater than concentrations of HCA or PAH molecules in cooked meats.^19^ The fact that the levels of dU and 8-oxo-dG increased strongly and prominently implies that both the deamination and oxidation of DNA were strongly accelerated during the cooking of food, which was exposed both to heat and ambient oxygen. Moreover, many other DNA lesions were also increased substantially in the foods. For example, 8,5′-cyclopurine-2′-deoxynucleosides were increased several-fold during roasting; these lesions are mutagenic and are documented to act as polymerase substrates in triphosphate form, although it is not yet known if phosphorylation occurs in cells.^20^ Interestingly, we found that DNA damage after cooking was considerably lower in potatoes than in pork and beef, suggesting that other components of plant tissues may confer substantial protection.

### Evidence That Damaged 2′-Deoxynucleosides Are Salvaged by Cells in Culture and Evoke DNA Damage and Repair Responses

**Figure 3:**
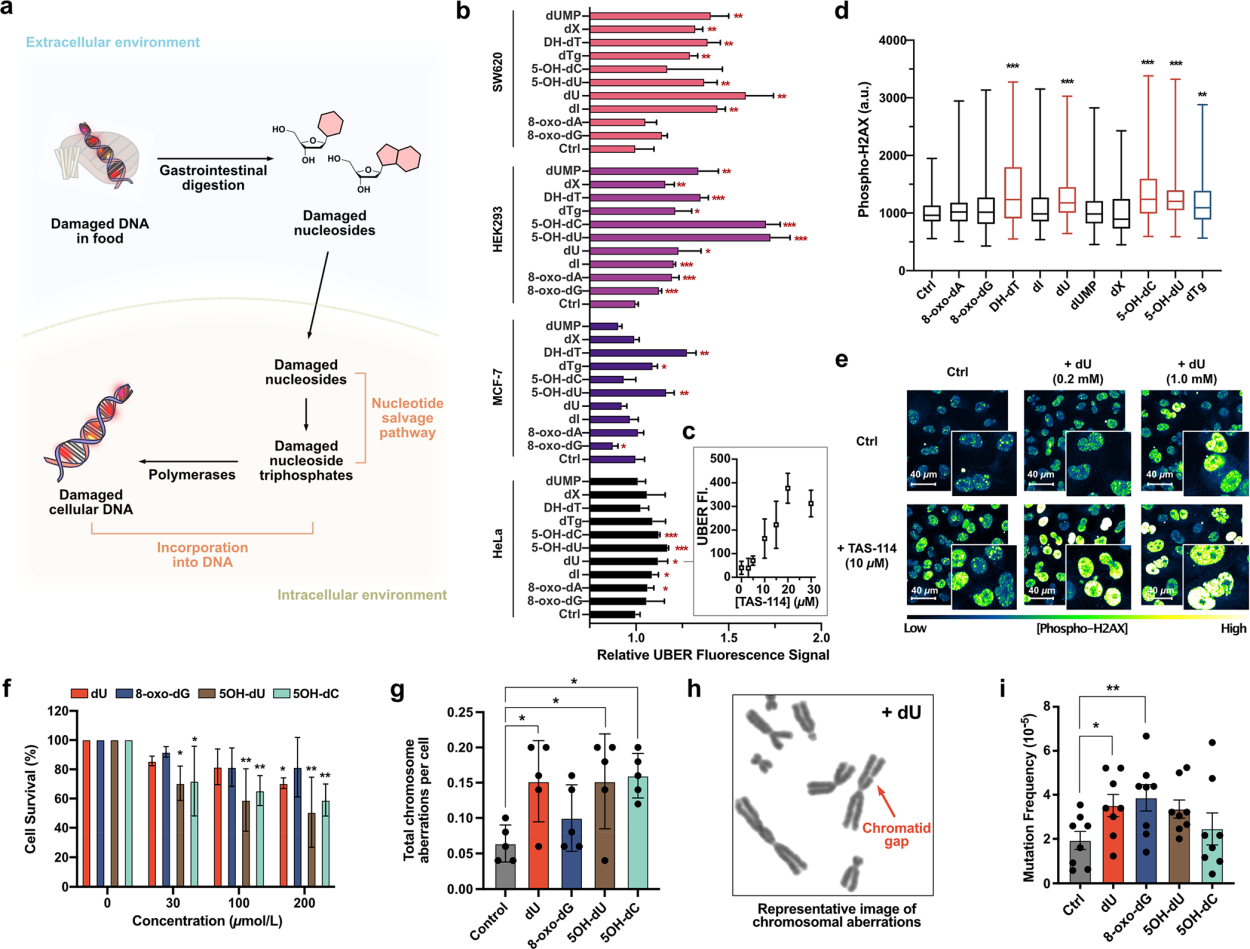
Cellular DNA damage responses to incubation with damaged 2′-deoxynucleosides. (a) Illustration of the pathway of damaged DNA in food to be incorporated into cellular DNA. (b) Flow cytometry results showing the relative fluorescence intensity of UBER in cells incubated with 200 μmol/L of damaged 2′-deoxynucleosides for 24 h, reflecting the BER activity of mitochondrial DNA. (c) Fluorescence intensity of UBER measured with flow cytometry in HeLa cells incubated with 200 μmol/L dU in the presence of a varied concentration of TAS-114 for 2 days. (d) Immuno-fluorescence intensity of γ-H2AX (a biomarker of DSB) in cells incubated with 200 μmol/L of damaged 2′-deoxynucleosides for 24 h. (e) Immunofluorescence images of γ-H2AX in cells incubated with dU and/or TAS-114, showing evidence of elevated DSB in the cells. (f) Cytotoxicity of damaged nucleosides in CHO cells measured by a colony formation assay (*N* = 4, *p ≤ 0.05, **p ≤ 0.01, ***p < 0.001 by Dunnett’s multiple comparisons test). (g) Chromosomal aberrations are elevated in CHO cells after incubation with 200 μmol/L damaged nucleosides for 24 h. *p ≤ 0.05. (h) Representative images of a chromatid break (gap), resulting from double-stranded DNA damage after exposure to 200 μmol/L dU (additional images in Supporting Information, Figure S7). (i) Evidence for the mutagenicity of damaged nucleosides after exposure to 200 μmol/L damaged nucleosides for 24 h, as measured by the HPRT mutation assay in CHO cells. *N* = 8, *p ≤ 0.05, **p ≤ 0.01, and ***p < 0.001 by Dunnett’s multiple comparisons test; uncertainties are standard deviations.

Following up on our findings that cooking markedly damages DNA in food, we next asked whether damaged DNA components pose risks to cells by acting as substrates for nucleotide salvage. Canonical DNA in food is ultimately digested into nucleosides by the gastrointestinal digestion system and then absorbed in the small intestine and transported into cells and circulation.^9,10^ Enzymes that are responsible for the nucleotide salvage pathway are known to exhibit imperfect selectivity, enabling the DNA uptake of modified nucleosides such as 5-bromo-2′-deoxyuridine (BrdU) and 5-ethynyl-2′-deoxyuridine (EdU) which are employed as markers of cellular DNA synthesis.^21^ We hypothesized that cooking-damaged DNA, digested into damaged 2′-deoxynucleosides upon consumption, might also be taken up into cellular DNA in a similar fashion (Figure 3a). The challenge in measuring the levels of damaged 2′-deoxynuclosides, if any, incorporated into cellular DNA is that the presence of such a lesion in genomic or mitochondrial DNA will be difficult to quantify directly, as it is being actively removed by repair pathways before it can be measured.

To bypass this issue, we initially measured the BER activity of cellular DNA evoked by the addition of damaged 2′-deoxynucleosides. As the forms of damaged 2′-deoxynucleosides studied here (except for the 8,5′-cyclopurine-2′-deoxynucleosides which are repaired by the nucleotide excision repair pathway) are known to be substrates for BER, the appearance of elevated BER activity implies the direct incorporation of damaged 2′-deoxynucleosides in the DNA.^20^

We employed a fluorescent probe specific to BER activity in cells (UBER) to gain evidence of cellular salvage and triphosphorylation, which are necessary for the incorporation of damaged 2′-deoxynucleosides into DNA. UBER binds covalently to AP sites in mitochondrial DNA (mtDNA) in intact cells and has been utilized for measuring mitochondrial BER responses to reactive oxygen species (ROS).^22^ While mitochondrial DNA lesions do not pose the direct cancer risks that those in genomic DNA do, the incorporation of damaged nucleosides into mtDNA would provide evidence for successful intracellular salvage and polymerase incorporation. The experiments included 10 different damaged 2′-deoxynucleosides (structures are shown in Supporting Information, Figure S4) and 4 cell lines (HeLa, MCF-7, HEK293, and SW620). We found that mitochondrial BER activity in cells increased in the presence of several of the damaged 2′-deoxynucleosides tested (Figure 3b), apparently as a result of defensive responses to increases in lesions in mtDNA.^23^ To further investigate the relationship between the enhanced DNA repair activity and salvage pathways that enable the incorporation of damaged nucleoside into DNA, we tested the effect of a chemical inhibitor of a nucleotide sanitization enzyme for one of the damaged components (dU).

TAS-114 is an inhibitor of dUTPase, which hydrolyzes dUTP into dUMP to prevent the misincorporation of dU into DNA.^24^ Inhibitor treatment in HeLa cells resulted in the further enhancement of BER signals in response to the incubation with dU in the cell culture medium (Figure 3c). This adds support to the notion that dU from external sources can be taken up via salvage and is iteratively phosphorylated to form the triphosphate analogue, enabling its incorporation into cellular DNA. Prior studies of dUTPase have suggested that it exists to address the hydrolysis of cytidine nucleotides that occurs directly in cells,^25^ while the new findings suggest that it can also prevent damage imported from external sources. Thus, the BER data for mitochondrial DNA support the notion of cellular salvage and uptake of damaged nucleosides, but further data were needed to assess any effects in chromosomal DNA.

A nucleotide gap in gDNA generated during the BER repair process is filled in by DNA polymerase β, and the resulting nick is sealed by ligase IIIα.^26^ However, high levels of lesions in DNA can accumulate, and multiple base excisions in clustered DNA lesions can result in proximal nucleotide gaps and nicks in both strands. This results in DSB, a serious form of DNA damage that can cause both chromosomal rearrangements and indel mutations.^26,27^ Thus, we assessed the levels of nuclear DSB after the incubation of damaged nucleosides with cells, employing phosphorylated histone H2AX (γ-H2AX) as a biomarker of cellular responses to DSB.^28^ The immunofluorescence assay results showed that levels of γ-H2AX were increased significantly in HeLa cells after incubation with 200 μmol/L of damaged nucleoside dU, 5-OH-dU, 5-OH-dC, DH-dT, or dTg for 24 h (Figure 3d). This result supports the hypothesis that damaged nucleosides were taken up into gDNA and subjected to high levels of BER there, ultimately resulting in DSB. To further confirm that DSB resulted from the metabolic activation of damaged nucleosides, the nucleotide sanitization pool was inhibited with TAS-114 in the presence of dU. Upregulated misincorporation of dU with TAS-114 exhibited much higher responses to DSB (Figure 3e), further supporting the involvement of the salvage pathway leading to BER and DSB when this form of damage is exposed to cells.

### Further Assessment of Genetic Damage after Exposure to High Levels of Damaged 2′-Deoxynucleosides

Given the above data documenting signals of mitochondrial base excision repair and chromosomal double-strand breaks in the DNA of cells incubated with damaged nucleosides, particularly for certain pyrimidines (Figure 3b,d), we tested the cytotoxicity of exposing damaged nucleosides to Chinese hamster ovary (CHO) cells using the colony formation assay (Figure 3f). Incubating the cells with 30–200 μM damaged pyrimidines dU, 5-OH-dU, and 5-OHdC for 8 days revealed significant cytotoxicity, while exposure to 8-oxo-dG did not. We performed further experiments to explicitly measure specific types of damage that may occur in chromosomal DNA upon exposure to high levels of damaged nucleosides. CHO cells were incubated for 24 h with 200 μmol/L dU, 8-oxo-dG, 5-OH-dU, and 5-OH-dC and then analyzed for chromosomal aberrations (after arresting the cells in the metaphase) compared to controls without added nucleoside. The data show an average ∼3-fold increase in chromosomal aberrations in the presence of the damaged pyrimidines dU, 5-OH-dU, and 5-OH-dC, including chromatid gaps, chromatid exchanges, and chromosomal rearrangements, while 8-oxo-dG showed little or no significant increase (Figure 3g,h and Supporting Information Figure S6). For chromosomal aberrations excluding gaps, the pyrimidines induced a yet larger 4-fold increase (Supporting Information Figure S6). We also evaluated possible mutagenic effects of the damaged nucleosides using a hypoxanthine phosphoribosyl transferase (HPRT) mutagenesis assay,^29^ which is commonly used to measure mutagenicity in mammalian cells, and two of the damaged nucleosides (dU and 8-oxo-dG) were found to induce statistically elevated levels (1.8- and 2.0-fold) of mutations (*p* = 0.0145 and 0.0062, Figure 3i), while 5-hydroxypyrimidines also showed average increases (∼1.7-fold) in mutagenicity but did not reach *p* > 0.05 (*p* = 0.085).

### Consumption of a Damaged 2′-Deoxynucleoside Contributes to DNA Damage in the Small Intestines of Rodents

**Figure 4:**
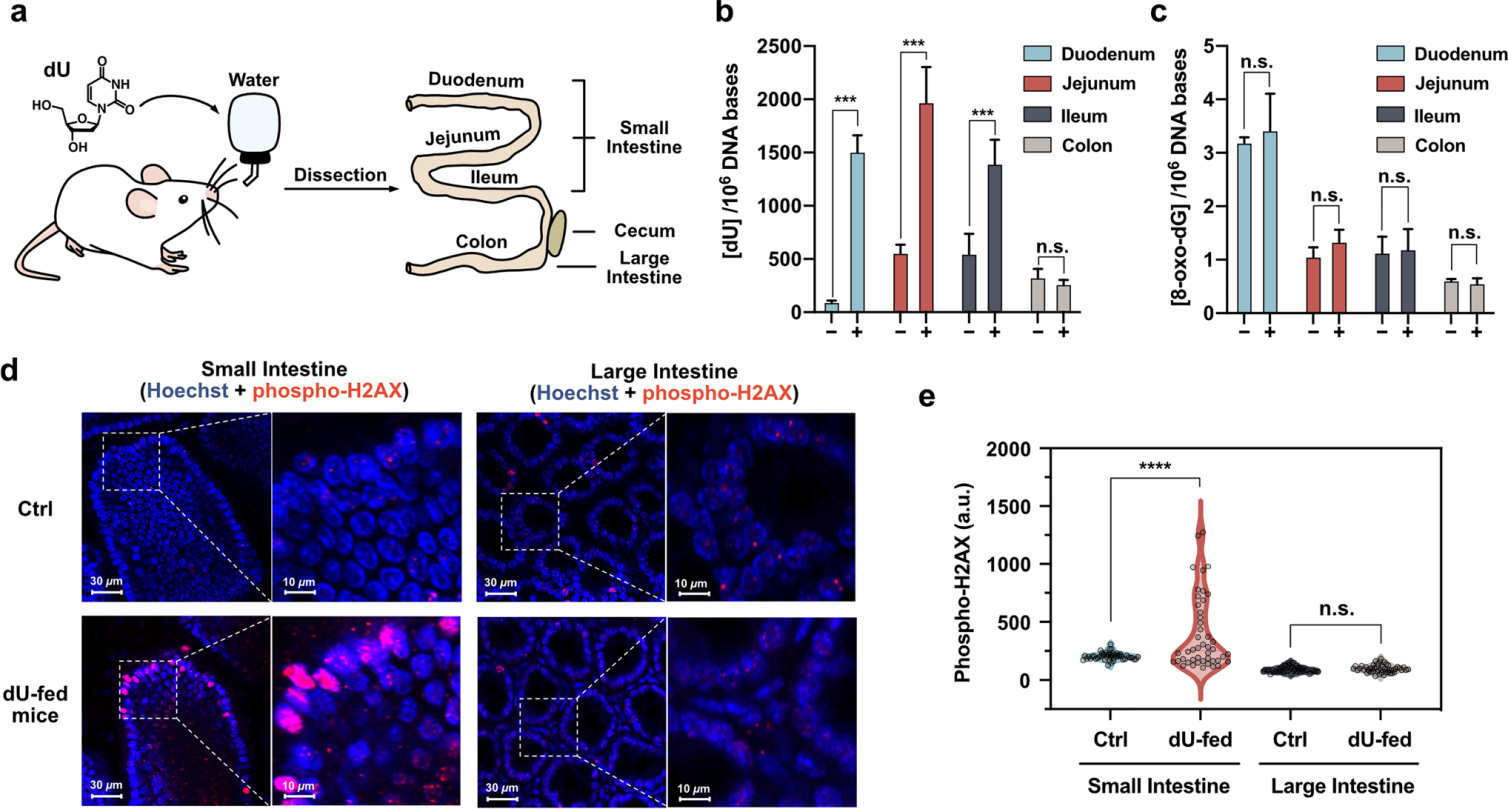
Adverse genetic effects of feeding high levels of a damaged nucleoside to mice. (a) Schematic illustration of oral feeding of dU to mice and analysis of intestinal tissue. LC–MS/MS quantification results of (b) dU and (c) 8-oxo-dG in gDNA extracted from the intestines of control (−) and dU-fed mice (+), showing 2.5-fold to 15-fold increases in levels of dU in the genomic DNA from these tissues. (d) Immunostaining of γ-H2AX in villi in the small intestine, showing enhanced DNA double-strand break (DSB) signals in response to dU feeding. Also shown are images of crypts in the large intestine. Tissues were costained with Hoechst 33343 (5 μg/mL) to highlight nuclear DNA. (e) Quantified intensities of γ-H2AX from red channels in panel d. Uncertainties are standard deviations (****p ≤ 0.0001) by the unpaired *t* test.

Given our observations that the cellular uptake of damaged 2′-deoxynucleosides can induce mitochondrial and genomic DNA damage in the cell culture, we pursued an animal model of this pathway to test whether damaged nucleosides that are orally consumed survive the digestive system and find their way into the DNA of tissues. As with animal studies of mutagenic small-molecule food species such as HCA and PAH, we employed high concentrations to observe maximal responses in a short span. We note that the concentration of damaged DNA used in these feeding experiments is in the same range of those used in prior PAH metabolite studies, while the amount of damaged DNA in food is calculated to be 3 to 4 order of magnitude higher than the metabolites.^30^ 2′-Deoxyuridine, the most abundant form of DNA damage caused by the cooking processes, was fed to mice (2 mg dU in 200 μL of PBS buffer daily) for a week through oral gavage (Figure 4a). After oral administration of dU, intestinal tissues (the site of absorption of canonical nucleosides) were examined for levels of damage in genomic DNA. From the tissue homogenates, gDNA was extracted and the levels of dU and 8-oxo-dG as a control were quantified with LC–MS/MS.

The results showed that dU was present at significantly higher levels in gDNA from the small intestines of dU-fed mice compared to that in control mice (Figure 4b). Increases were substantial, with an increase of up to 2000 dU per million gDNA bases in the duodenum and jejunum, corresponding to 15-fold and 3.5-fold increases, respectively. Note that these elevated levels were observed in the presence of presumably intact DNA repair pathways in the mice and thus are likely lower than actual initial uptake. DNA incorporation levels of dU in the ileum were significant (2.5-fold increase), albeit smaller than in the earlier digestive tract and nonexistent in colon tissue, consistent with prior studies showing that canonical 2′-deoxynucleosides are primarily absorbed earlier in the digestive tract.^31^ It seems possible that the absence of villi in the colon resulted in a negligible incorporation of dU. Given that colorectal cancer is more frequent than small intestinal cancer in the clinic, further studies are needed regarding this localization. For control experiments, the level of 8-oxo-dG (not fed in the experiment) was measured in mouse intestinal DNA, showing no significant difference between the two, confirming that dU feeding caused no oxidation of dG (Figure 4c). The results confirm that the increased level of dU in gDNA of dU-fed mice resulted from direct DNA incorporation of the damaged component rather than by indirectly inducing ROS (which may also increase the deamination of dC).^32^

We further employed the γ-H2AX immunostaining assay to measure DSB in mouse intestinal tissues after a week of oral administration of dU. Microscopic images of the stained intestinal tissue showed that the level of γ-H2AX was significantly higher in epithelial cells of villi in small intestines from dU-fed mice than that of control mice (Figure 4d,e). In contrast, mice fed with 2 mg of dC, the canonical 2′-deoxynucleoside precursor of dU, daily for a week showed no observable enhancement in DSB levels, implying that the imbalance of the nucleotide pool is not a chief cause of these signals (Supporting Information, Figure S8). Taken together, our results suggest that dU in the diet may be taken up in enterocytes of villi of the small intestine after consumption and then enters the intracellular salvage pathway followed by incorporation into cellular DNA.

At least at the high concentrations tested here, this results in elevated DNA repair responses leading to increased DSB. The results document that an orally ingested damaged 2′-deoxynucleoside, generated at high levels in food DNA during cooking, potentially survives the digestive system and find its way into cellular DNA, leading to a serious form of DNA damage in intestinal tissues.

### Damaged DNA Can Be Digested and Incorporated into Cellular DNA upon Consumption

**Figure 5:**
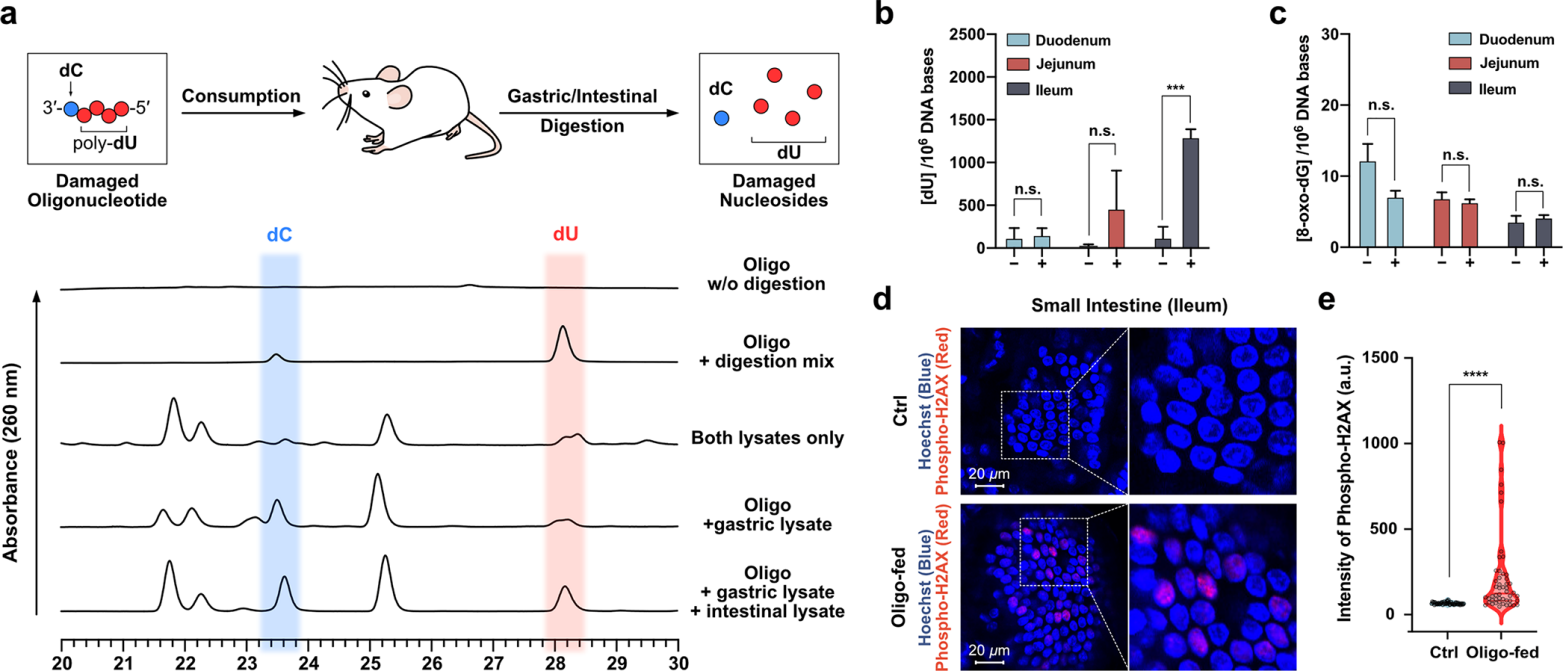
(a) HPLC analysis of the *in vitro* digestion of 10 μg of a damaged oligodeoxynucleotide (5′-d(UUUUC)-3′) with a digestive enzyme mix, gastric lysate, or gastrointestinal lysate at 37 °C for 24 h. (b) Levels of dU and (c) control 8-oxo-dG in gDNA extracted from the intestines of control (−) and damaged DNA-fed mice (+), showing >10-fold increases in levels of dU in the genomic DNA from later small intestinal tissue. (d) Immunostaining of γ-H2AX in villi of the small intestine (ileum), showing enhanced DNA double-strand break (DSB) signals (in red) in response to damaged oligonucleotide feeding for 1 week. (e) Quantification results of red fluorescence (level of γ-H2AX) measured from epithelial cells in panel d. ****p ≤ 0.0001 by the unpaired *t* test. Tissues were co-stained with blue Hoechst 33343 (5 μg/mL) to highlight nuclear DNA. Uncertainties are standard deviations.

The above experiments evaluated the effects of feeding a deaminated nucleoside monomer to rodents; however, our hypothesis requires that the ingestion of DNA or DNA fragments containing such damage is followed by processing by nuclease and phosphatase activities in the stomach, gut, and cells to the nucleotide/nucleoside form. Although this has been established for canonical DNA, it has not yet been tested with damaged DNA to our knowledge. To test this, we synthesized an oligodeoxynucleotide (5′-d(UUUUC)-3′) containing four dU residues, and *in vitro* digestion of the oligodeoxynucleotide by a commercial digestive enzyme mix was analyzed with HPLC (Figure 5a). We found that the oligodeoxynucleotide was digested completely into free 2′-deoxynucleotides (dU and dC). As a second test with native digestive enzymes, lysates were prepared from the homogenized stomach and small intestines of mice, including gastric and intestinal juices. HPLC analysis clearly showed that the damaged oligodeoxynucleotide was digested, releasing the corresponding 2′-deoxynucleosides in the presence of both gastric and intestinal lysates. Thus, the damaged DNA base (even with consecutive substitution) does not prevent the digestion of DNA containing it.

Finally, we tested whether directly feeding DNA containing deaminated bases would lead to the observable incorporation of damage into intestinal tissue. Mice were fed the above synthetic oligodeoxynucleotide (2 mg in 200 μL of PBS daily) for 7 days, and the DNA extracted from intestinal tissue was then analyzed for damage content. The results showed that the level of dU in the extracted mouse gDNA was significantly increased (>10-fold) in the later part of the small intestine upon the consumption of the damaged oligodeoxynucleotide, while the level of 8-oxo-dG as a control remained unchanged (Figure 5b,c). Considering that the digestion of the damaged oligodeoxynucleotide requires digestive enzymes in the small intestine (Figure 5a), lesser absorption/incorporation into the early part of the small intestine may plausibly reflect the requirement for complete digestion during the transit of the intestine. Also, consistent with the above experiments, small intestine tissues showed increased level of DSB after the feeding of the damaged DNA (Figure 5d,e).

## Conclusions

Our data represent, to our knowledge, the first documentation of damage to food DNA as a result of cooking and suggest a possible new etiology for genetic risk from cooked foods. Our findings add support to previous conclusions that high-temperature cooking confers a significant health risk with frequent and long-term consumption.^1,2^ However, the new data suggests the possibility that a significant portion of the pathologic genetic dysfunction from cooked foods may plausibly arise from the consumption of food DNA itself, along with the previously identified small-molecule metabolites. As pointed out above, the consumption of cooked beef or pork in a meal can easily involve the ingestion of at least 1 g of DNA.^9^ Our findings suggest that an estimated 6 × 10^17^ dU nucleotides (0.3 mg/1.0 μmol) and substantial quantities of other damaged 2′-deoxynucleotides may be ingested with 100 g of red meat mildly roasted for 20 min. This is as much as 3 to 4 orders of magnitude higher than the amounts of activated small-molecule metabolites such as HCAs and PAHs that occur in cooked food;^19^ moreover, the damage resulting from salvaged 2′-deoxynucleosides (if incorporated via polymerases) is direct and requires no chance reaction with DNA. We do not discount the genetic risks that reactive small molecules in foods pose; indeed, these two mechanisms are not mutually exclusive.

Clearly, this initial study is very early, and establishing this hypothesized connection firmly will require more follow-up studies in toxicology. In addition, although our mechanistic hypothesis of salvaging damaged nucleosides is supported by several lines of evidence here (particularly for dU), we cannot yet rule out some unforeseen indirect mechanism whereby exposure to the damaged monomers found in cooked food elevates cellular DNA damage and subsequent repair responses.

It is likely that different cooking methods and diverse foods will result in large variations in DNA damage in the food. Our experiments revealed distinct differences in the level of damage by types of cooking, with roasting (220 °C) causing more damage than boiling (100 °C) relative to raw foods. Extended times at elevated temperatures have an important effect, as shown by our studies of DNA incubated at 95 °C over time. We note that our roasting procedure was relatively mild, and higher-temperature cooking methods (grilling, frying) and long times (smoking) are common in public use. More work is needed to test the effects of varied cooking procedures.

In the current studies, DNA from potatoes was substantially less damaged than was that from meats; the reason for this is not yet clear, although we speculate that the presence of high levels of starch may contribute to some protection against reactive oxygen species, perhaps by scavenging free radicals.^33^ It remains to be seen if this holds true for other plant foods. Also potentially relevant is the fact that most plants are known to contain far smaller amounts of DNA per weight compared to animals (Supporting Information, Table 1).^9^ The observation that plant-based diets^34^ are association with lower cancer risks would also be consistent with these findings; further studies are required to better understand DNA damage in cooked plant-based foods relative to meats.

Many forms of damage are observed directly in cellular DNA, and cells have evolved numerous repair enzymes and pathways to address them. However, cells also present a line of defense against DNA damage even before it occurs in DNA, in the form of nucleotide pool sanitation enzymes.^35^ We have observed that dU and 8-oxo-dG were the two most abundant forms of DNA damage in food emerging during cooking processes, and cells possess nucleotide sanitation enzymes (e.g., dUTPase and MTH1)^15^ to specifically address deaminated and oxidized 2′-deoxynucleoside triphosphates. Prior studies have cited these enzymes’ function to defend against spontaneous deamination and oxidation that arise intracellularly during normal metabolism. We suggest the additional possibility that their activities are also crucial to defending against the consumption and salvage of damaged DNA components from food. Indeed, we show that the suppression of dUTPase activity markedly increases levels of DNA damage in the presence of dU in the medium (Figure 3e). Certain human populations are known to possess genetically attenuated nucleotide sanitization activities or DNA repair activities,^36^ and the new findings, if confirmed more broadly, suggest that high-temperature cooking may pose yet more serious risks to these individuals, especially with frequent consumption over years. Future population studies will be helpful in establishing such a connection.

Taken together, our experiments suggest a possible novel mechanism that has the potential to help explain connections between high-temperature cooking (particularly of meats) and human cancers and metabolic diseases. The results prompt the need for further studies to assess the effects of long-term exposure at lower concentrations to determine which specific damaged DNA species are of greatest concern. If additional studies support these early findings, then this suggests new reasons to emphasize food preparation at reduced temperatures and times as well as the consumption of vegetables and raw foods in general.

Finally, we note that, in addition to possible relevance to diet, the observation of the salvaging of damaged nucleosides into cells and tissues may serve as a useful tool in future studies of DNA damage and repair. Typically, researchers employ general mechanisms (such as adding oxidizing species to the medium) to induce cellular DNA damage, resulting in the formation of several species simultaneously. The proposed nucleoside salvage mechanism suggests the possibility of introducing specific damaged species one at a time into cells; future work will explore this possibility.

## Supporting Information Available

Detailed experimental procedures (PDF)
Transparent Peer Review report available (PDF)
