# Supplementary material for: Possible Genetic Risks from Heat-Damaged DNA in Food
Source: ACS Cent Sci. 2023 Jun 1;9(6):1170–9. doi: 10.1021/acscentsci.2c01247 (PMC10311654; doi:10.1021/acscentsci.2c01247)
Supplement: Supplementary file 1 — oc2c01247_si_001.pdf [file oc2c01247_si_001.pdf]

Supporting Information

Possible Genetic Risks from Heat-Damaged DNA in Food

Yong Woong Jun[1,6], Melis Kant[2], Erdem Coskun[2,3], Takamitsu A. Kato[4], Pawel Jaruga[2], Elizabeth Palafox[1], Miral Dizdaroglu[2], Eric T. Kool[1,5]*

[1]Department of Chemistry, Stanford University, Stanford, CA, 94305, USA

[2]Biomolecular Measurement Division, National Institute of Standards and Technology, Gaithersburg, MD, 20899, USA

[3]Institute for Bioscience & Biotechnology Research, University of Maryland, Rockville, MD 20850, USA

[4]Department of Environmental & Radiological Health Sciences, Colorado State University, Fort Collins, CO, USA

[5]Sarafan ChEM-H, Stanford Cancer Institute, Stanford University, Stanford, CA 94305, USA

[6]Present Address: Department of Chemistry, Korea Advanced Institute of Science and Technology (KAIST), Daejeon 34141, Republic of Korea

Table of Contents

Materials and Methods 2
- Instrumentation 2
- Materials 2
- Uracil quantification from gDNA extracted from cells 2
- DNA extraction from food 2
- Measurement of DNA damage in gDNA 3
- Measuring mitochondrial DNA repair activity in cells 3
- Immunostaining of γ-H2AX in cells 3
- Colony formation assay 4
- Chromosomal aberration analysis 4
- Hypoxanthine phosphoribosyl transferase (HPRT) mutation analysis 4
- Oral administration of deoxyuridine or damaged oligodeoxynucleotide to mice 4
- Measurement of the levels of dU and 8-oxo-dG from mouse intestinal tissue 4
- Immunostaining of γ-H2AX in mouse intestines 5
- Preparation of gastrointestinal lysates from mice 5
- Analysis of *in vitro* digestion of a damaged oligonucleotide 5

Tables 9

References 10

Materials and Methods

Instrumentation

For flow cytometry, fluorescence intensities were measured on a Quanteon instrument with a blue laser 488 nm (100 mW), and B530 detector (530/30 nm) with 552LP mirror in Stanford shared FACS facility. Fluorescence images were recorded on an inverted Zeiss LSM 780 multiphoton laser scanning confocal microscope in Cell Science Imaging Facility (CSIF) at Stanford. Fluorescence emission spectra were recorded on a Fluoroskan Ascent microplate reader. Products of DNA damage were identified and quantified by GC-MS/MS and LC-MS/MS with isotope dilution using stable isotope-labeled analogues of the products as internal standards.

Materials

Uracil-DNA glycosylase (UDG) was purchased from New England Biolabs (M0280S). UBERGreen dye was synthesized as described previously,[1] or was purchased from Skunkworks Biosciences (Palo Alto, CA, USA). Ground beef (80 %lean), ground pork (80 %lean), and potatoes were purchased from Whole Foods Market (Cupertino, CA, USA). DNeasy mericon Food Kit was purchased from QIAGEN (69514). Easy-DNA gDNA purification kit was purchased from Thermo Fisher Scientific (K180001). TAS-114 (HY-124062) and TH-588 (HY-12814) were purchased from Med. Chem. Express. Damaged nucleosides used for cellular experiments were synthesized[2] or purchased from Biosynth Carbosynth. Phospho-Histone H2AX (Ser 139) Rabbit mAb was purchased from Cell Signaling Technology (9718S). This was visualized with Goat anti-Rabbit IgG secondary antibody, Alexa Fluor Plus 647 (Invitrogen, A32733). Plastic feeding tubes (20 G, 30 mm) were purchased from Instech (50-475-764). 2′-Deoxyuridine-$^{13}C_9$,$^{15}N_2$ was purchased from Cambridge Isotope Laboratories, Inc. (Tewksbury, MA, USA). The internal standards ThyGly-$^{2}H_4$, FapyAde-^{13}C,$^{15}N_2$, and FapyGua-^{13}C,$^{15}N_2$ used for GC-MS/MS measurements and 8-oxo-dG-$^{15}N_5$ used for LC-MS/MS measurements are a part of the NIST (National Institute of Standards and Technology) Standard Reference Material 2396 Oxidative DNA Damage Mass Spectrometry Standards (NIST SRM 2396) (http://www.nist.gov/srm/index.cfm and https://www-s.nist.gov/srmors/view_detail.cfm? srm=2396). The internal standards *R*-cdA-$^{15}N_5$, *S*-cdA-$^{15}N_5$, *S*-cdG-$^{15}N_5$ and *R*-cdG-$^{15}N_5$ used for LC-MS/MS measurements were synthesized and isolated as described.[3-4] Material Transfer Agreement was granted between Stanford and NIST (IC2021-1936).

Uracil quantification from gDNA extracted from cells

HeLa human cervical tumor cells were cultured in DMEM supplemented with FBS (10 %), penicillin (100 U/mL), and streptomycin (100 U/mL) in a humidified incubator at 37 °C with 5 %CO_2. The cells were passaged when they reached approximately 80 % of confluency. gDNA was extracted from cells by using Easy-DNA gDNA Purification Kit (ThermoFisher, K180001). Briefly, 5×10^6 cells were prepared in 200 µL PBS in a microfuge tube, then mixed with lysis solution and incubated at 65 °C for 10 min. After extraction with chloroform, DNA/RNA was precipitated by adding 0.1× *v/v* 3 mol/L NaOAc buffer (pH 5), 1 µg/mL glycogen, and 3.75× *v/v* chilled ethanol, then kept at -80 °C for 2 h. The DNA/RNA pellets were obtained by centrifuging the tubes at 21000 RCF for 60 min in a cold rood (ca. 4 °C). RNA was removed by adding RNase for 30 min at 37 °C, then pure gDNA was precipitated by adding 0.1× v/v 3 mol/L NaOAc buffer (pH 5), 1 µg/mL glycogen, and 3.75× chilled ethanol, then kept at -80 °C for 2 h. The DNA pellets were obtained by centrifuging the tubes at 21000 RCF for 60 min in a cold rood (ca. 4 °C). The DNA pellet was dissolved in 20 mmol/L Tris-HCl buffer (pH 8) containing 1 mmol/L DDT and 1 mmol/L EDTA, then heated at 95 ˚C in a sealed tube up to 7 days. To measure the level of uracil, 40 U/mL UDG (NEB, M0280S) enzyme, and 100 µmol/L UBER were added to the heated gDNA and incubated overnight at 37 °C. gDNA was precipitated by adding 0.1× *v/v* 3 M NaOAc buffer (pH 5), 1 µg/mL glycogen, and 3.75× chilled ethanol, then kept at -80 °C for 2 h. The DNA pellets were obtained by centrifuging the tubes at 21000 RCF for 60 min in a cold rood (ca. 4 °C). The precipitated gDNA was re-dissolved with the buffer, and the fluorescence intensity was measured with a Fluoroskan plate reader (emission collected at 538 nm with excitation at 485 nm).

DNA extraction from food

Ground beef (80 %lean), ground pork (80 %lean), and potatoes purchased from Whole Foods Market were prepared in three ways (raw, boiled, or roasted). For boiling process, each food was boiled in boiling water (ca 100 °C) for 20 min. For the roasting process, a standard kitchen oven was pre-heated to 218 °C (425 °F) and food samples were roasted for 15 min. DNA in the ground beef and pork was extracted with a DNeasy Maricon Food Kit. Briefly, the food was homogenized by using a blade, then mixed with Food lysis buffer and proteinase K and incubated at 60 ˚C

for 30 min. After cooling to room temperature on ice, the mixture was centrifuged, then the supernatant was extracted with chloroform. DNA was purified by using QIAquick spin column provided in the kit. DNA isolation yield of the kit for potatoes was not high enough to secure enough amount of DNA for DNA damage analysis by HPLC. DNA from potatoes was isolated by using CTAB buffer.[5] Briefly, homogenized potatoes were mixed with CTAB buffer (20 g/L CTAB, 1.4 mol/L NaCl, 0.1 mol/L Tris/HCl, and 20 mmol/L EDTA), then incubated at 65 °C for 30 min. After centrifuging for 10 min at 12000 g, the supernatant was extracted with chloroform, then precipitated with CTAB precipitation buffer (5 g/L CTAB, and 0.04 mol/L NaCl). The precipitated pellet was extracted with chloroform, then the pure DNA was precipitated out with isopropyl alcohol.

Measurement of DNA damage in gDNA

Aliquots of 50 μg of three independently isolated DNA samples from each beef, pork and potato samples were used for GC-MS/MS and LC-MS/MS measurements. ThyGly, FapyAde and FapyGua were measured by GC-MS/MS using ThyGly-$^{2}H_4$, FapyAde-^{13}C,$^{15}N_2$, and FapyGua-^{13}C,$^{15}N_2$, respectively, as their internal standards, whereas LC-MS/MS was used for the measurement of 8-oxo-dG, *R*-cdA, *S*-cdA, *S*-cdG and *R*-cdG using 8-oxo-dG-$^{15}N_5$, *R*-cdA-$^{15}N_5$, *S*-cdA-$^{15}N_5$, *S*-cdG-$^{15}N_5$ and *R*-cdG-$^{15}N_5$, respectively, as their internal standards. Hydrolysis of DNA samples to release these products from DNA as free bases or 2′-deoxynucleosides, and other measurement conditions have been described in detail elsewhere.[6] 2′-Deoxyuridine in hydrolyzed DNA samples was measured by LC-MS/MS using dU-$^{13}C_9$,$^{15}N_2$ as the internal standard using the same experimental conditions as for the measurement of the other 2′-deoxynucleosides. The mass/charge transitions used for these measurements were *m/z* 229 → *m/z* 113 for dU and *m/z* 240→ *m/z* 119 for dU-$^{13}C_9$,$^{15}N_2$.

Measuring mitochondrial DNA repair activity in cells

HeLa human cervical cancer cells, MCF-7 human breast cancer cells, HEK293, and SW620 colorectal cancer cells were cultured in DMEM supplemented with 4 g/L D-glucose, L-glutamate, 110 mg/L sodium pyruvate, 10 %FBS, 0.1 U/L penicillin, and 0.1 U/L streptomycin in a humidified incubator at 37 °C with 5 %CO_2. The cells were passaged when they reached approximately 80 % of confluency. The cells were seeded onto 12 well cell culture plates at the calculated confluency for 4.0×10^5 cells at measurement. The cell culture medium was removed, then subsequently replacing the medium with the same cell culture medium containing 200 µmol/L of damaged nucleosides (8-oxo-dG, 8-oxo-dA, dI, dU, 5-OH-dU, 5-OH-dC, Tg, DH-dT, dX, or dUMP) and 10 µmol/L UBER, and the cells were incubated for 24 h at 37 °C in the presence of 5 %CO_2 in the air (In the case of TAS-114 inhibition test, the cells were pre-incubated with different concentration of TAS-114 for 24 h, then after the addition of UBER and dU the cells were incubated another 24 h at 37 °C). The medium was removed, and the cells were washed with PBS. After the cells were trypsinized with 200 µL trypsin for 5 min at 37 °C, the cells were transferred to a 1.5 mL tube and neutralized with 200 µL cell culture medium. The cells were centrifuged at 500 × g for 5 min, then the supernatant was removed. The cell pellets were dispersed in 200 µL PBS buffer, then after the solutions were transferred to a 5 mL polystyrene round-bottom tube, the fluorescence intensity of UBER was measured in a flow cytometer with blue 488 nm laser and 530 nm emission filter. The FACS data were analyzed and processed with FlowJo software.

Immunostaining of γ-H2AX in cells

HeLa human cervical tumor cells were cultured in DMEM supplemented with 4 g/L D-glucose, L-glutamate, 110 mg/L sodium pyruvate, 10 %FBS, 0.1 U/L penicillin, and 0.1 U/L streptomycin in a humidified incubator at 37 °C with 5 %CO_2. The cells were passaged when they reached approximately 80 % of confluency. The cells were seeded onto 6-well cell culture plates at the calculated confluency for 1.0×10^6 cells at measurement. The cells were incubated with 200 µmol/L damaged nucleosides (8-oxo-dG, 8-oxo-dA, dI, dU, 5-OH-dU, 5-OH-dC, dTg, DH-dT, dX, or dUMP). The cell growth media was removed, then the cells were washed with PBS buffer three times. The cells were fixed with 4 %formaldehyde (methanol free) for 10 min at rt. After washing the cells with PBS three times, the cells were permeabilized with 0.5 %Triton-X in PBS for 5 min at rt. After washing with PBS once, the cells were blocked with 3 %BSA in PBS for 1 h at rt. After the block solution was removed, γ-H2AX (Ser129) primary antibody diluted in PBS with 1 %BSA was added to the cells and incubated for 24 h at 4 °C. The solution was removed, and the cells were washed with PBS for three times, then the secondary antibody (Alexa 647) diluted in PBS with 1 %BSA was added and incubated for 1 h at rt. After washing the cells with PBS for three times, the cells were mounted on the confocal microscope, and imaged using a 633 nm laser while the emission was collected at 650 nm to 700 nm. The acquisition of images and calculation of intensity were done in ImageJ software.

Colony formation assay

To investigate the cytotoxicity of damaged nucleosides, a colony formation assay was carried out. Cells were treated with varied concentrations (as described) of nucleosides continuously during colony formation. Cells were fixed with 100 %ethanol and stained with crystal violet solution 8 days after the incubation period. Colonies were observed under a light microscope and colonies containing more than 50 cells were counted as survivors.[7] Cell survival assays were carried out a minimum of three times, independently.

Chromosomal aberration analysis

Damaged nucleoside-induced cytogenetic damage was investigated in metaphase-spread chromosomes. Cells were treated with 200 μmol/L of nucleoside for 24 h. In the last 6 h, Colcemid was added to induce metaphase arrest. Cells were harvested during metaphase, trypsinized and then suspended in 4 mL of 75 mmol/L KCl solution warmed to 37 °C and placed in a 37 °C water bath for 20 min. A Carnoy's solution (3:1 methanol to acetic acid) was added to the samples according to the standard protocol.[8] Fixed cells were dropped onto slides and allowed to dry at room temperature. Slides were stained with 5 %Giemsa solution in Gurr buffer. Chromosomal aberration analysis was carried out under a Zeiss Axioskop microscope. Fifty metaphase cells were analyzed for chromosomal aberration analysis. Three independent experiments were performed.

Hypoxanthine phosphoribosyl transferase (HPRT) mutation analysis

Prior to use in the HPRT mutation assay, CHO cells were grown with HAT supplement (2×10^{-4} mol/L hypoxanthine, 2×10^{-7} mol/L aminopterin, and 1.75×10^{-5} mol/L thymidine) for two days to reduce the level of spontaneous HPRT mutations. Then, the cells were recovered in regular media for two days. After incubating cells, 200 μmol/L of damaged nucleoside was added for 24 h. After compound treatment, single-cell suspensions were diluted and grown in a nonselective medium for a period of time sufficient to allow phenotypic expression prior to plating for determination of mutant frequency. Phenotypic expression time is approximately 7 days for HPRT mutation. Then, 3×10^5 cells were plated in P60 cell culture dishes with a selective agent, 6-thioguanine (6-TG, 5 μg/mL). Cells from corresponding dishes were also plated at a low density (300 cells/mL) in the absence of the selective agent to determine plating efficiency. All cell cultures were incubated for 10 days, permitting a sufficient growth period prior to scoring the colonies. Each HPRT mutation experiment was carried out 4 times to ensure reproducibility.[7, 9]

Oral administration of 2′-deoxyuridine or damaged oligodeoxynucleotide to mice

C57BL/6J mice (4 weeks old) were purchased from The Jackson Laboratory (000664), and caged in the Stanford animal facility. Deoxyuridine purchased from Biosynth Carbosynth (ND06282) was diluted with PBS to prepare 10 mg/mL solution. The deaminated oligodeoxynucleotide (5′-d(UUUUC)-3′) was purchased from IDT. The mice were fed with 200 μL of the solution (2 mg dU or 2 mg of the oligodeoxynucleotide) though oral gavage with plastic feeding tubes (20 G, 30 mm, Instech, 50-475-764). The feeding of a 2′-deoxynucleoside or an oligodeoxynucleotide was performed once per day for seven days. All experimental procedures were conducted in accordance with protocols approved by Stanford University's Institutional Animal Care and Use Committee (IACUC, assurance number A3213-01).

Measurement of the levels of dU and 8-oxo-dG from mouse intestinal tissue

Mice fed with dU or damaged oligodeoxynucleotide for a week were euthanized with carbon dioxide followed by cervical dislocation. Intestines were removed from mice, washed with PBS, then cut into 4 sections (duodenum, jejunum, ileum, and colon). Digested solids inside the intestines were flushed out with PBS buffer using a plastic feeding needle. The tissue samples were placed in a mortar, then ground into a powder with a pestle in the presence of liquid nitrogen. This homogenization process was repeated twice to obtain a fine powder. gDNA was extracted from the homogenized tissue samples with Easy-DNA gDNA Purification Kit (ThermoFisher, K180001). LC-MS/MS was used to measure dU and 8-oxo-dG in DNA samples as described above. All experimental procedures were conducted in accordance with protocols approved by Stanford University's Institutional Animal Care and Use Committee (IACUC, assurance number A3213-01). This work with the Project No. MML-16-0016 was reviewed and approved by the Research Protections Office of NIST.

Immunostaining of γ-H2AX in mouse intestines

Mice were euthanized and intestinal tissues isolated and flushed as above. The tissues were fixed with 4 %formaldehyde for 30 min at rt by filling the solution in the intestines. After washing the tissue with PBS for three times, the tissues were opened through a lateral incision and mounted on a slide glass. Then the tissue was permeabilized with 0.5 %Triton-X in PBS for 20 min. The permeabilization buffer was removed and washed with PBS, then the tissue samples were blocked with a block solution (10 % BSA in PBS) for 2 h at rt. After removing the solution, the tissue was washed with PBS buffer and treated with phosphor-H2AX primary antibody diluted in PBS buffer containing 0.3 %Triton-X and 1% BSA for 1 h at 37 °C. After the staining solution was removed, the tissue was washed with PBS for three times. The secondary antibody (Alexa 647) in PBS containing 0.3 %Triton-X and 1 %BSA was added to the tissue, and incubated for 1 h at 37 °C. The stained tissue was washed with PBS three times with 5 min incubation each. The tissues were then stained with 1 µg/mL Hoechst for 30 min then imaged on the confocal microscope equipped with 405 and 633 nm laser sources. The acquisition of images was done in ImageJ software. For the quantification of fluorescence intensity, the emission from red channel was collected from over 50 epithelial cells from each image. All experimental procedures were conducted in accordance with protocols approved by Stanford University's Institutional Animal Care and Use Committee (IACUC, assurance number A3213-01).

Preparation of gastrointestinal lysates from mice

Mice were euthanized and stomach and small intestine tissue were removed. After homogenizing without flushing to include the gastric and intestinal juice inside with scissors on ice, it was further homogenized by using gentleMACS dissociator. The lysates were prepared from the sample by following the previously reported procedure.[10]

Analysis of *in vitro* digestion of a damaged oligonucleotide

10 µg of a damaged oligodeoxynucleotide (5′-d(UUUU)C-3′) purchased from IDT was subjected to digestion with Nucleoside Digestion Mix purchased from NEB following the manufacturer's instructions, or with extracted gastrointestinal lysates for 24 h at 37 °C (70 µL of 0.5 µg/µL concentration). The digested nucleosides were analyzed with HPLC using authentic dU or dC to confirm peak identity. Eluent: 1 % of methanol in 10 mmol/L ammonium acetate buffer (pH 4.5) was gradually increased to 30 %methanol over 32 min during the analysis.

Disclaimer

Certain equipment, instruments, or materials, commercial or non-commercial, are identified in this manuscript in order to specify the experimental procedure adequately. Such identification is not intended to imply recommendation or endorsement of any product or service by NIST, nor is it intended to imply that the materials or equipment identified are necessarily the best available for the purpose.

Figures

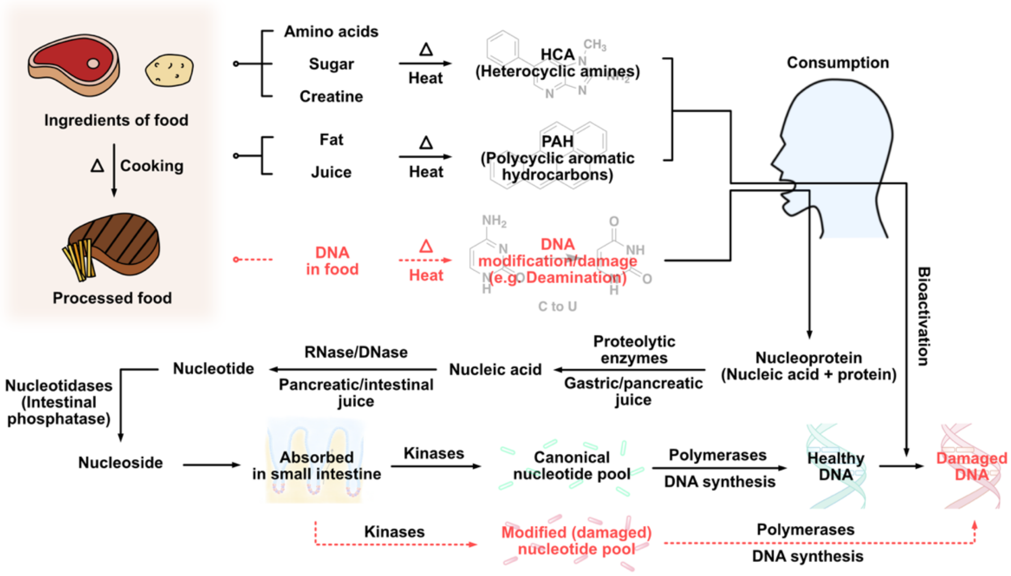

Figure S1. Gastrointestinal digestion pathway of DNA.

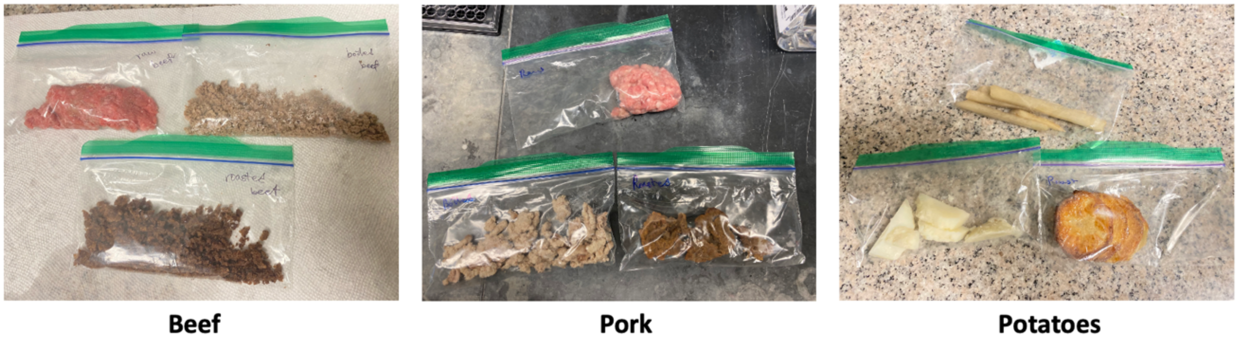

Figure S2. Images of raw and processed beef, pork, and potatoes used in the study.

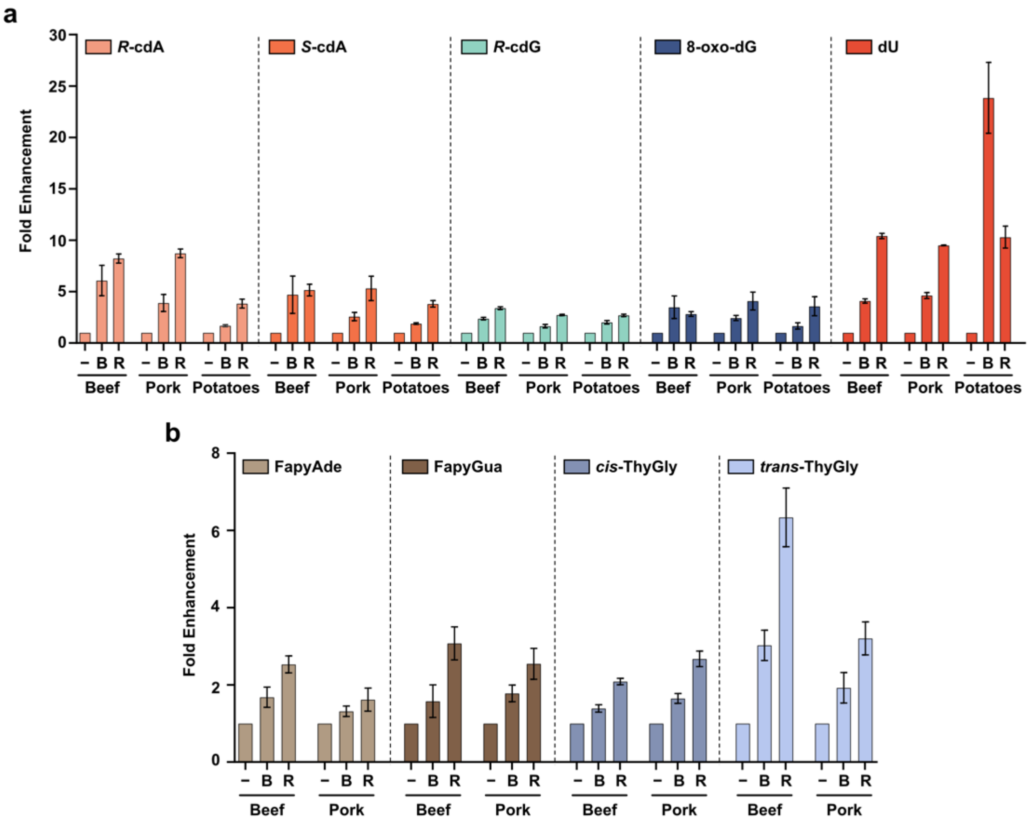

Figure S3. Changes in levels of DNA damage on cooking (relative to uncooked foods): (a) *R*-cdA, *S*-cdA, *R*-cdG, 8-oxo-dG, and dU, and (b) FapyAde, FapyGua, *cis*-ThyGly, and *trans*-ThyGly in beef, pork, and potatoes that are raw or heat-processed in relative amounts. Uncertainties are standard deviations.

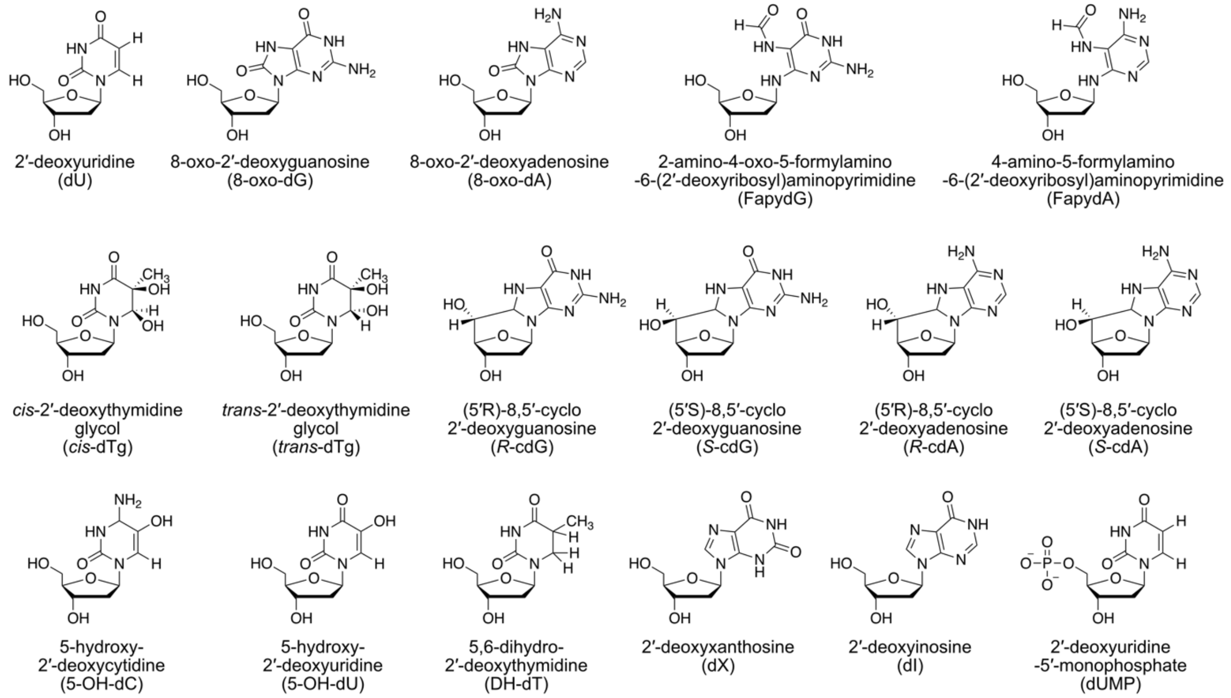

Figure. S4. Chemical structures of the damaged 2′-deoxynucleosides studied along with names and abbreviations

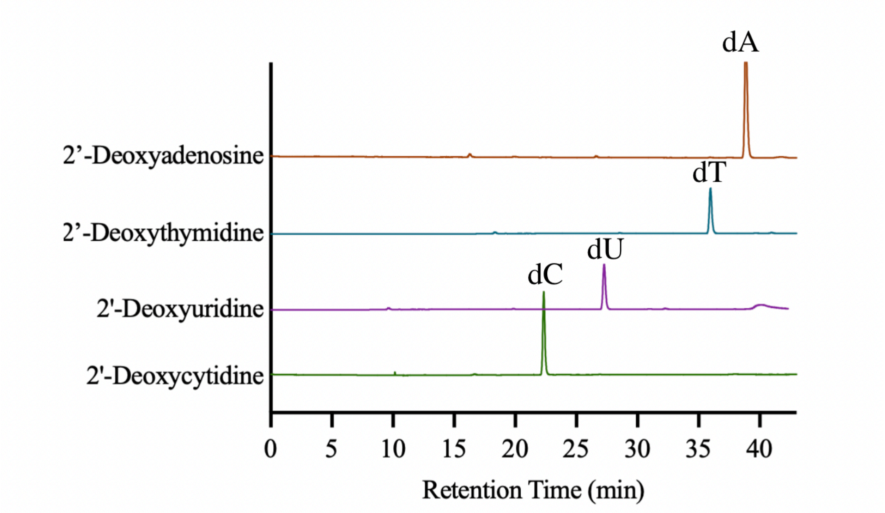

Figure S5. Retention time of reference nucleosides.

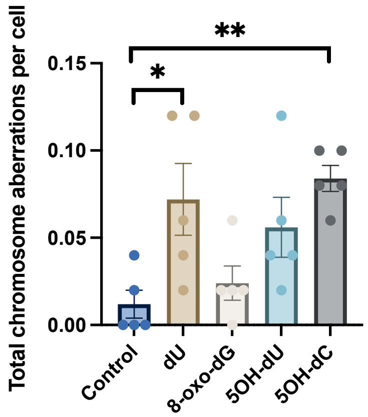

Figure S6. Total chromosomal aberrations in CHO cells excluding chromatid gaps after incubation with 200 μmol/L of damaged nucleoside for 24 h. N=5; error bars show standard deviations. *p≤0.05, **p≤0.01, ***p<0.001 by Dunnett's multiple comparisons test.

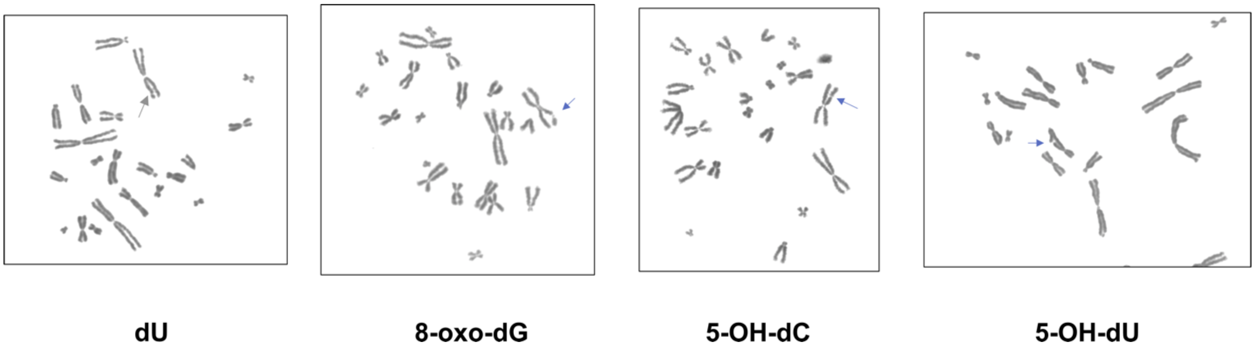

Figure S7. Representative images of chromatid aberrations in metaphase spreads of chromosomal DNA from CHO cells resulting from double stranded DNA damage after exposure to 200 μM of damaged 2′-deoxynucleosides for 24 h. Arrows highlight chromatid breaks.

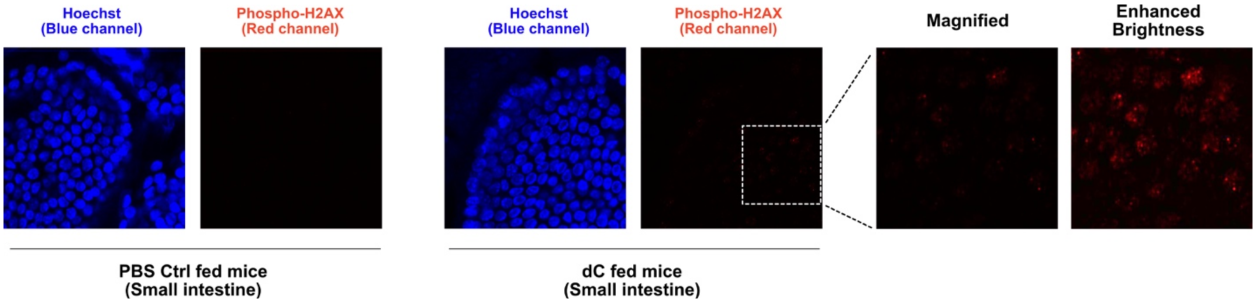

Figure S8. Control mouse feeding experiments. Mice were fed with either only PBS or 2 mg of dC in PBS daily for a week. Representative images are shown of immunostaining of γ-H2AX in villi in small intestine, showing the level of DNA double-strand break (DSB) signals in response to feeding dC. Tissues were co-stained with Hoechst 33343 (5 μg/mL). The results show that feeding a canonical 2′-deoxynucleoside that might cause imbalance in nucleotide pools has a negligible effect on the level of DSB. N=3 animals in each arm; at least 3 tissue samples were examined for each animal. Magnified and brightness-enhanced images by 100% on the right show that the images are not blank, but weakly fluorescent.

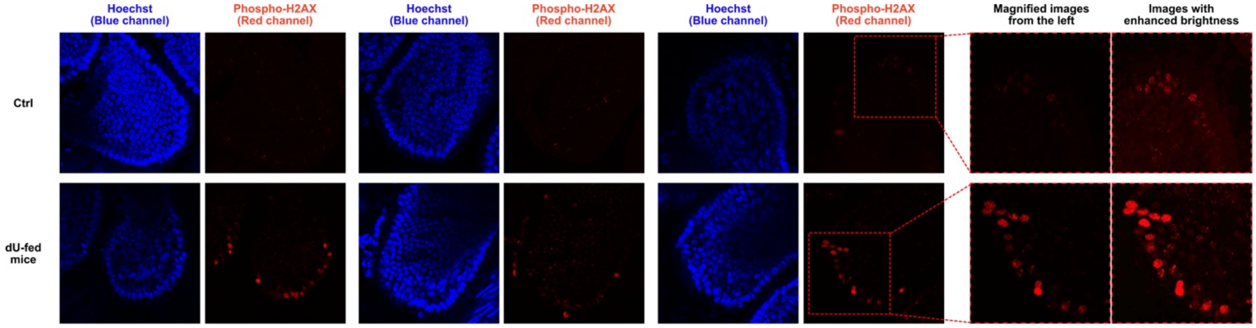

Figure S9. Immunostaining of γ-H2AX in villi in the small intestine of mice, showing enhanced DNA double-strand break (DSB) signals in response to dU feeding. Also shown are images of crypts in the large intestine. Tissues were co-stained with Hoechst 33343 (5 μg/mL) to highlight nuclear DNA. Images of the right are magnified and brightened by 100%, showing the relative fluorescence intensities from DSB.

Tables

Table S1. Temperatures of common cooking procedures.

Cooking Method	Temperature (℃)
Broiling	260 to 288
Roasting	≧ 205
Grilling	121 to 232
Deep frying	175 to 190
Baking	163 to 177
Pressure cooking	≈121
Boiling	100
Steaming	100
Smoking	93 to 105
Sous vide	45 to 85

References

1. Wilson, D. L.; Kool, E. T., Ultrafast Oxime Formation Enables Efficient Fluorescence Light-Up Measurement of DNA Base Excision. *J. Am. Chem. Soc.* **2019,** *141*, 19379–19388.
2. Barvian, M. R.; Greenberg, M. M., Diastereoselective Synthesis of Hydroxylated Dihydrothymidines Resulting from Oxidative Stress. *J. Org. Chem.* **1993,** *58*, 6151–6154.
3. Jaruga, P.; Birincioglu, M.; Rodriguez, H.; Dizdaroglu, M., Mass Spectrometric Assays for the Tandem Lesion 8,5‘-cyclo-2‘-Deoxyguanosine in Mammalian DNA. *Biochemistry* **2002,** *41*, 3703–3711.
4. Birincioglu, M.; Jaruga, P.; Chowdhury, G.; Rodriguez, H.; Dizdaroglu, M.; Gates, K. S., DNA Base Damage by the Antitumor Agent 3-Amino-1,2,4-Benzotriazine-1,4-Dioxide (Tirapazamine). *J. Am. Chem. Soc.* **2003,** *125*, 11607–11615.
5. Lipp, M.; Brodmann, P.; Pietsch, K.; Pauwels, J.; Anklam, E., IUPAC Collaborative Trial Study of a Method to Detect Genetically Modified Soy Beans and Maize in Dried Powder. *J. AOAC Int.* **1999,** *82*, 923–928.
6. Kant, M.; Jaruga, P.; Coskun, E.; Ward, S.; Stark, A. D.; Baumann, T.; Becker, D.; Adhikary, A.; Sevilla, M. D.; Dizdaroglu, M., Ne-22 Ion-Beam Radiation Damage to DNA: From Initial Free Radical Formation to Resulting DNA-Base Damage. *ACS Omega* **2021,** *6*, 16600–16611.
7. Haskins, J. S.; Su, C.; Maeda, J.; Walsh, K. D.; Haskins, A. H.; Allum, A. J.; Froning, C. E.; Kato, T. A., Evaluating the Genotoxic and Cytotoxic Effects of Thymidine Analogs, 5-Ethynyl-2′-Deoxyuridine and 5-Bromo-2′-Deoxyurdine to Mammalian Cells. *Int. J. Mol. Sci.* **2020,** *21*, 6631.
8. Kato, T. A., Human Lymphocyte Metaphase Chromosome Preparation for Radiation-Induced Chromosome Aberration Analysis. In *Methods Mol. Biol.*, Springer: 2019; pp 1–6.
9. Little, J. B.; Nagasawa, H.; Pfenning, T.; Vetrovs, H., Radiation-Induced Genomic Instability: Delayed Mutagenic and Cytogenetic Effects of X Rays and Alpha Particles. *Radiat. Res.* **1997,** *14,*, 299–307.
10. Jun, Y. W.; Wilson, D. L.; Kietrys, A. M.; Lotsof, E. R.; Conlon, S. G.; David, S. S.; Kool, E. T., An Excimer Clamp for Measuring Damaged-Base Excision by the DNA Repair Enzyme NTH1. *Angewandte Chemie* **2020,** *132*, 7520–7525.
