# Supplementary material for: Possible Genetic Risks from Heat-Damaged DNA in Food
Source: ACS Cent Sci. 2023 Jun 1;9(6):1170–9. doi: 10.1021/acscentsci.2c01247 (PMC10311654; doi:10.1021/acscentsci.2c01247)
Supplement: Supplementary file 2 — oc2c01247_si_002.pdf [file oc2c01247_si_002.pdf]

oc-2022-01247v.R1

Name: Peer Review Information for "Possible Genetic Risks from Heat-Damaged DNA in Food"

First Round of Reviewer Comments

Reviewer: 1

Comments to the Author

The manuscript “Possible Genetic Risks from Heat-Damaged DNA in Food” by Jun et al. describes the damage to DNA caused by cooking food at high temperatures as well as the metabolic fate of these damaged products when they are fed to cells. This is an area of science likely to be of high public health interest due to the widespread consumption of heated (e.g. microwaved) food products. Given the possibility of sensationalist stories this work could give rise to (“New study finds microwaved food causes cancer”) the authors are careful to couch their experimental findings properly, including in the title (“Possible risks”) and throughout the manuscript (“We emphasize that this overall hypothesis cannot be proven in such an initial study, etc”). The authors are responsible scientists and I expect that, if published, they will take care to make sure the important message of the study is not overinterpreted. Regarding the science, the study is thorough, and the conclusions are measured and supported by the data. As such, I have only a few comments I believe should be addressed prior to publication.

1. The sentence, “As with animal studies of mutagenic small molecule food species such as HCA and PAH, we employed high concentrations to observe maximal responses in a short span.” should be supported by references to studies that use similarly high concentrations of mutagens in animal studies.

2. Figure 3f does not appear to be discussed in the main text.

3. In general information on some statistical tests is missing, particularly from the CHO experimental data (figures 3f-g, S6)

4. Related: the discussion states “an average ≈3-fold increase in chromosomal aberrations in the presence of the damaged pyrimidines dU, 5-OH-dU, 5-OH-dC, including chromatid gaps, chromatid exchanges, and chromosomal rearrangements, while 8-oxo-dG showed little or no significant increase”. Is this effect statistically significant for dU?

5. Similarly: the discussion states: “the mutagenicity of 5-hydroxypyrimidines rising to statistical significance after one day of exposure”; However, Fig 3i shows statistical calculations for only dU and 8-oxo-dG, not for the 5-hydroxypyrimidines. This should be addressed.

Reviewer: 2

Comments to the Author

The prevailing model regarding harmful effects induced by consumption of over-heated food has been that the reactive small-molecule agents generated during high-temperature cooking process induce DNA alkylation, resulting in genome damage. The authors in this paper aim to test the less well-recognized/-studied hypothesis that damaged nucleos(t)ides derived from cooked food that can be uptaken by cells and in turn cause genome damage, are also an important causative factor. They deployed both in vitro biochemical/cell-based and in vivo mice models to test this hypothesis. Furthermore, the topic is also of relevance to multiple research fields, and interesting for the broader readership. I thus think that overall this study is potentially a good fit for this journal. However, I list below my main concerns why I cannot recommend the manuscript in its current form, for publication in ACS Cent Sci.

I hope that the authors will do their due diligence to address these concerns and ensure all conclusions are backed up by concrete (and direct) evidence, by revised and/or new experimentation reflecting a more real-world relevant context. I would be happy to review a fully-revised version of the paper.

- A human cell repairs over ~10,000 DNA lesions per day, so I wonder if the damaged base-pairs brought into the cell can mount sufficiently significant impacts
- Using BER activity to report on damaged nucleotide incorporation to host DNA is not a reliable approach, since there are just too many factors/variables that could lead to BER activity modulation in a given cellular context following treatment with potential mutagens. These non-natural nucleot(s)ides could also have multiple adverse effects on genome replication / biogenesis / metabolic pathways. Furthermore, if one is using an indirect reporter, one does need to know how much upregulation is really functionally significant: e.g., can we responsibly consider 1.2 – 1.7 fold as sufficient to elicit a functional impact? To validate that this indirect report is a reliable way to assess incorporation, the authors should first knockdown (if knockout is non-viable) BER in host cells, and quantitatively evaluate the extent of incorporation. (The use of pharmacological inhibitors could result in pleotropic effects and thus results from genetic knockdown (using 3 different knockdown lines and 3 dfferent control lines, to rule out well-known off-target effects with sh/siRNAs, would help us to draw unambiguous conclusions of these data).
- How much genotoxicity do these damaged-DNAs/nucleos(t)ides induce to the cells/animals under study? Tox profiling, ames test, alama-blue cell viability assays under the same experiment conditions, etc., should be performed. (g-H2AX upregulation results that the authors showed could be elicited by multiple causes, such as triggering programmed cell-death, for example)
- The authors should do competition studies in the presence of the appropriate ratios of natural to damaged nucleos(t)ides since incorporation of modified nucleos(t)ides (or in this context, damaged nucleos(t)ides), is easily outcompeted (or well-protected) by native nucleos(t)ides: evidenced by, for instance, success shown in EdU labeling pulse-chase experiments and Par-Clip techniques.
- Should their overall hypothesis of the DNA in the cooked food inducing health risks hold true, then the authors should expect there would be some level of evolutionary selection pressure.

For instance, do nucleotide importers have selective mutations common only to humans and not in for instance, to Neandarthals (or to our next closest ancestors such as chimpanzees), who/that did (do) not eat cooked food...?

- In the mice feeding studies, the feeding regimen should be more reflective of normal prandial consumption such as concentration of potential mutagen balanced with natural DNA/nucleos(t)ide intake.
- Furthermore, as it is well known that natural nucleotide pool imbalance would lead to cellular stress, it would be pertinent for the authors to perform side-by-side comparative assessment of the extent of g-H2AX upregulation seen in intestinal tissue between, feeding natural nucleosides (e.g., dA) and the authors' claimed mutagen, under otherwise identical conditions. It appears that upregulation observed in Fig 4/5-d remains modest, and feeding of an excess of natural nucleos(t)ides on a daily basis over a week-long period to mice could result in the same phenotype. Note: The authors should also show more frames of images in supporting information and quantify the image data to compare/contrast respective fold magnitude of upregulation.
- As it is common for all biological investigations and data reported in decent peer-reviewed journals, data analysis should be consistently accompanied by proper statistical treatment: for instance, t-test or one-way ANOVA and corrected t-test, as applicable in all main and SI data plots. Otherwise it is hard for readers to evaluate whether or not relative differences seen across different nucleos(t)ides are statistically significant. They should clearly and consistently state in figure legends no. of biological replicates, sample size, and all other commonly-expected relevant parameters.

Minor: I would recommend slight change in title: currently it is a bit misleading as it sounds like the cellular DNA has been damaged by heat… (perhaps 'from food' is clearer)

Author's Response to Peer Review Comments:

STANFORD UNIVERSITY, Stanford, California 94305

Eric T. Kool
kool@stanford.edu
George and Hilda Daubert Professor

Tel 650/724-4741
Fax 650/725-0259

Prof. Editor
Senior Editor
ACS Central Science

March 20, 2023

Dear Dr. Editor,

With this letter are submitting our revised manuscript entitled "Possible Genetic Risks from Heat-Damaged DNA in Food". We appreciate the reviewers' careful reading of the work and their constructive comments. In response, we have carried out multiple new lines of experiments and revised the manuscript substantially. Below is a detailed list of reviewer comments and our responses:

# Reviewer: 1

The manuscript "Possible Genetic Risks from Heat-Damaged DNA in Food" by Jun et al. describes the damage to DNA caused by cooking food at high temperatures as well as the metabolic fate of these damaged products when they are fed to cells. This is an area of science likely to be of high public health interest due to the widespread consumption of heated (e.g. microwaved) food products. Given the possibility of sensationalist stories this work could give rise to ("New study finds microwaved food causes cancer") the authors are careful to couch their experimental findings properly, including in the title ("Possible risks") and throughout the manuscript ("We emphasize that this overall hypothesis cannot be proven in such an initial study, etc"). The authors are responsible scientists and I expect that, if published, they will take care to make sure the important message of the study is not overinterpreted. Regarding the science, the study is thorough, and the conclusions are measured and supported by the data. As such, I have only a few comments I believe should be addressed prior to publication.

We thank the reviewer for the appreciation of our work and for the helpful comments.

Q1. The sentence, "As with animal studies of mutagenic small molecule food species such as HCA and PAH, we employed high concentrations to observe maximal responses in a short span." should be supported by references to studies that use similarly high concentrations of mutagens in animal studies.

We thank the reviewer. We have now added a citation supporting this. LeBlanc *et. al.* (BMC Genomics, 2022, 542) thoroughly studied the genotoxicity of benzo-[a]-pyrene (BaP, a representative compound of PAH) using duplex sequencing. In the study, mice were fed with 50 mg/kg BaP for 28 days by oral gavage, which is a similar dose as ours but with 3× longer period of time; while the average concentration of BaP in cooked foods is 3–4 magnitudes less than the concentration of damaged DNA. Note that although one example is cited, this high dose strategy is common in the field. This is now also mentioned in the text explicitly.

Q2. Figure 3f does not appear to be discussed in the main text.

Thanks for pointing this out; we have now added a short description of the figure in the main text.

Q3. In general information on some statistical tests is missing, particularly from the CHO experimental data (figures 3f–g, S6)

We have now performed additional experiments to increase replicates in those experiments to better evaluate confidence levels, and statistical test results are now included.

Q4. Related: the discussion states "an average ≈3-fold increase in chromosomal aberrations in the presence of the damaged pyrimidines dU, 5-OH-dU, 5-OH-dC, including chromatid gaps, chromatid exchanges, and chromosomal rearrangements, while 8-oxo-dG showed little or no significant increase". Is this effect statistically significant for dU?

Yes, it is statistically significant, and this is now explicitly stated in the revised text. The cells treated with dU showed statistically significant increase in both of chromosomal aberration ($p=0.0192$) and mutation frequency ($p=0.0145$).

Q5. Similarly: the discussion states: "the mutagenicity of 5-hydroxypyrimidines rising to statistical significance after one day of exposure"; However, Fig 3i shows statistical calculations for only dU and 8-oxo-dG, not for the 5-hydroxypyrimidines. This should be addressed.

In our assessment including new replicates, the average mutagenicity of 5-hydroxypyrimidines was increased (~1.7-fold) but did not achieve $p>0.05$ in the total experiments ($p=0.085$). The statement has been revised in the main text to make clear what is statistically significant.

# Reviewer: 2

The prevailing model regarding harmful effects induced by consumption of over-heated food has been that the reactive small molecule agents generated during high-temperature cooking process induce DNA alkylation, resulting in genome damage. The authors in this paper aim to test the less well-recognized/-studied hypothesis that damaged nucleos(t)ides derived from cooked food that can be uptaken by cells and in turn cause genome damage, are also an important causative factor. They deployed both in vitro biochemical/cell-based and in vivo mice models to test this hypothesis. Furthermore, the topic is also of relevance to multiple research fields, and interesting for the broader readership. I thus think that overall this study is potentially a good fit for this journal. However, I list below my main concerns why I cannot recommend the manuscript in its current form, for publication in ACS Cent Sci. I hope that the authors will do their due diligence to address these concerns and ensure all conclusions are backed up by concrete (and direct) evidence, by revised and/or new experimentation reflecting a more real-world relevant context. I would be happy to review a fully-revised version of the paper.

We thank the reviewer for noting the relevance of this work and for the helpful comments.

Q1. A human cell repairs over ~10,000 DNA lesions per day, so I wonder if the damaged base-pairs brought into the cell can mount sufficiently significant impacts

As the reviewer notes, the capacity of DNA repair system is the key to assess the actual risk of this hypothesis. There are three main defensive mechanisms that can protect genomic integrity against this genotoxic pathway; (i) selectivity of kinases and polymerases toward canonical ones, (ii) nucleotide sanitization enzymes, and (iii) glycosylases initiating BER process to repair the incorporated damage. What we observed from the feeding experiments is

that the consumption of dU at high doses can be salvaged and can accumulate in gDNA even in the presence of intact DNA repair systems, showing that the DNA repair system can be overwhelmed by the influx of damaged DNA.

DNA repair systems only rarely miss damage, which is fortunate for human populations. However, as they do occasionally fail, this leads to significant cancer rates in large populations over decades. As the reviewer understands, in real human diets the levels of damage (mg/kg) will be lower than studied here. However, countering these low levels is the lifetime of exposure over the population and the cumulative nature of oncogenesis. This first study cannot address such long-term, low-dose effects, but rather asks whether the overall mechanism is biochemically reasonable, at least at high doses. We have carefully added cautions to the text to note this:

> Clearly, this initial study is very early, and establishing this hypothesized connection firmly will require more follow-up studies in toxicology, involving varied doses, extended times, and further comparisons of different damaged nucleosides. For example, it is not yet known whether low doses of damaged DNA or nucleosides over long times would still have deleterious effects. Further, dilution of damaged DNA components with undamaged ones could potentially have a mitigating effect on genotoxicity, which also remains to be studied.

and in the introduction:

> We emphasize that this overall hypothesis cannot be proven in such an initial study. Indeed, studies of small-molecule agents such as PAH and HCA in cooked foods have proceeded over decades, and risks to humans are only seen in large population studies. Thus, our goal is to test the individual parts of the food DNA hypothesis, which may lead to insights into its feasibility.

Q2. Using BER activity to report on damaged nucleotide incorporation to host DNA is not a reliable approach, since there are just too many factors/variables that could lead to BER activity modulation in a given cellular context following treatment with potential mutagens. These non-natural nucleot(s)ides could also have multiple adverse effects on genome replication / biogenesis / metabolic pathways. Furthermore, if one is using an indirect reporter, one does need to know how much upregulation is really functionally significant: e.g., can we responsibly consider 1.2 – 1.7 fold as sufficient to elicit a functional impact? To validate that this indirect report is a reliable way to assess incorporation, the authors should first knockdown (if knockout is non-viable) BER in host cells, and quantitatively evaluate the extent of incorporation. (The use of pharmacological inhibitors could result in pleotropic effects and thus results from genetic knockdown (using 3 different knockdown lines and 3 different control lines, to rule out well-known off-target effects with sh/siRNAs, would help us to draw unambiguous conclusions of these data).

As the reviewer points out, assessing the adverse effects of damaged nucleosides by only a single reporter may not be reliable enough. That is why we have investigated the effects of damaged nucleosides not only on BER activity but also on other genotoxic characteristics using four additional measurements: double strand breaks (DSB) by immunostaining of gamma-H2AX, cell viability measurements, assessment of chromosomal aberrations, and mutation frequency. In sum, results from multiple different assays show the consistent genotoxic effects of damaged nucleosides.

The validation of the indirect reporter using knockdown cell lines and inhibitors which are suggested by the reviewer has already been studied thoroughly in our previous paper which developed the assay (Jun et. al., Angew. Chem. Int. Ed. 2022, 61, e202111829).

Q3. How much genotoxicity do these damaged-DNAs/nucleos(t)ides induce to the cells/animals under study? Tox profiling, ames test, alama-blue cell viability assays under the same experiment conditions, etc., should be performed. (g-H2AX upregulation results that the authors showed could be elicited by multiple causes, such as triggering programmed cell-death, for example)

We have carried out genotoxicity assays in collaboration with an expert in toxicology (co-author Takamitsu A. Kato) using the HPRT mutation assay, which is widely used to test mutagenicity in mammalian cells (revised Figure 3i). The new data, carried out with additional replicates to increase confidence levels, show that treating cells with dU nearly doubled the mutation frequency in CHO cells. (see revised Fig 3i for mutation analysis and chromosomal aberrations (Fig. S6), both with additional replicates).

We assure the reviewer that we recognize the importance of larger animal studies for the long term, and are in the midst of planning a large collaboration to follow up on these early findings with sequencing-based mutagenesis studies in animals, and tumor formation studies in mice. We expect to publish those data in the future.

Q3. The authors should do competition studies in the presence of the appropriate ratios of natural to damaged nucleos(t)ides since incorporation of modified nucleos(t)ides (or in this context, damaged nucleos(t)ides), is easily outcompeted (or well-protected) by native nucleos(t)ides: evidenced by, for instance, success shown in EdU labeling pulse-chase experiments and Par-Clip techniques.

This is an important question to assess the actual long-term risk of consuming damaged DNA, as normal diet contains high level of canonical nucleosides which compete with damaged ones toward incorporation. To address the reviewer's comment, we have now pointed out the possible competition effect as an added caution in the text (see Q1 above). Like the reviewer, we are also highly interested in assessing the actual risk of this hypothesized pathway at "normal" DNA/damage levels, but it is beyond the scope of this work as it requires months/years of feeding studies with a large population. This current work aims to perform initial tests of the feasibility of this new hypothesis, as described in the main text.

Q4. Should their overall hypothesis of the DNA in the cooked food inducing health risks hold true, then the authors should expect there would be some level of evolutionary selection pressure. For instance, do nucleotide importers have selective mutations common only to humans and not in for instance, to Neandarthals (or to our next closest ancestors such as chimpanzees), who/that did (do) not eat cooked food...?

We enjoyed this very interesting comment, as we also have had similar discussions. It turns out that early humans have been cooking food for ca. 100K years, and one does wonder whether humans might have evolved different repair enzyme levels relative to (for example) apes. Although no comparison data seem yet available, that would be an interesting future area of research to pursue.

Q5. In the mice feeding studies, the feeding regimen should be more reflective of normal prandial consumption such as concentration of potential mutagen balanced with natural DNA/nucleos(t)ide intake.

Please see Q1 for reviewer 1 and Q3 for reviewer 2.

Q6. Furthermore, as it is well known that natural nucleotide pool imbalance would lead to cellular stress, it would be pertinent for the authors to perform side-by-side comparative assessment of the extent of g-H2AX upregulation seen in intestinal tissue between, feeding natural nucleosides (e.g., dA) and the authors' claimed mutagen, under otherwise identical conditions. It appears that upregulation observed in Fig 4/5-d remains modest, and feeding of an excess of natural nucleos(t)ides on a daily basis over a week-long period to mice could result in the same phenotype.
Note: The authors should also show more frames of images in supporting information and quantify the image data to compare/contrast respective fold magnitude of upregulation.

More frames of images for the immunostaining experiments are now added to supporting information (Supplementary Figure S9), and the quantification analysis of the images is now included in the main text and figures.

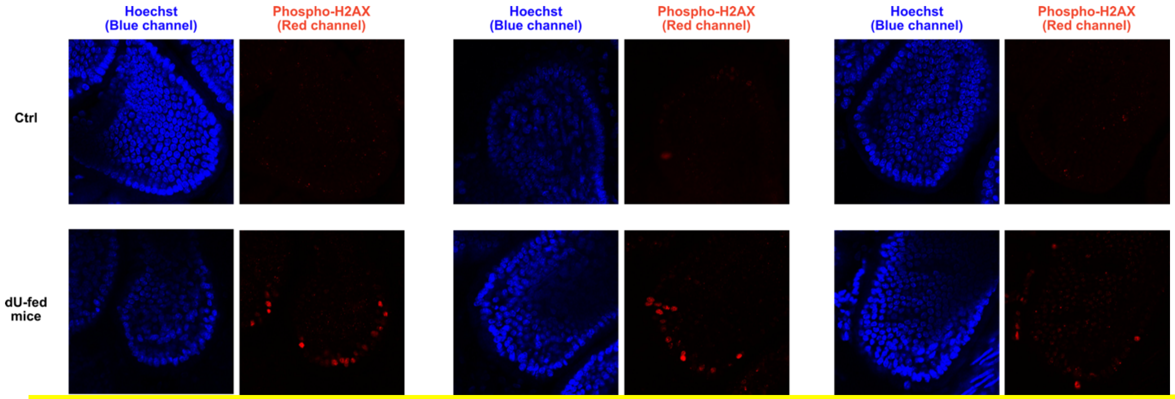

Figure S9. Immunostaining of γ-H2AX in villi in the small intestine of mice, showing enhanced DNA double-strand break (DSB) signals in response to dU feeding. Also shown are images of crypts in the large intestine. Tissues were co-stained with Hoechst 33343 (5 µg/mL) to highlight nuclear DNA.

The reviewer's point is well taken. In the revised manuscript, we carried out further mouse feeding experiments to test whether the nucleotide imbalance caused by feeding a canonical nucleoside (dC) results in increased DSB in intestinal tissues (Supplementary Figure S8). The results showed that the feeding of dC induced no discernible enhancement in the level of DSB, which implies that the nucleotide pool imbalance which can be caused by feeding a nucleoside is not likely the cause of the increased level of DSB observed in dU feeding experiments. This result is now included in the main text.

We further employed the γ-H2AX immunostaining assay for measuring DSB in mouse intestinal tissues after a week of oral administration of dU. Microscopic images of the stained intestinal tissue showed that the level of γ-H2AX was significantly higher in epithelial cells of villi in small intestines from dU-fed mice than that of control mice (Figure 4d,e). In contrast, mice fed with 2 mg of dC, the canonical nucleoside precursor of dU, daily for a week showed no observable enhancement in DSB levels, implying that the imbalance of the nucleotide pool is not a chief cause of these signals (Suppl. Figure S8).

Q7. As it is common for all biological investigations and data reported in decent peer-reviewed journals, data analysis should be consistently accompanied by proper statistical treatment: for instance, t-test or one-way ANOVA and corrected t-test, as applicable in all main and SI data plots. Otherwise it is hard for readers to evaluate whether or not relative differences seen across different nucleos(t)ides are statistically significant. They should clearly and consistently state in figure legends no. of biological replicates, sample size, and all other commonly-expected relevant parameters.

We have now included all the suggested statistical information.

Sincerely,

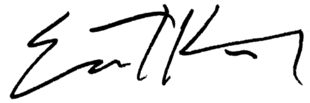

Eric T. Kool
Department of Chemistry
Stanford University

oc-2022-01247v.R2

Name: Peer Review Information for "Possible Genetic Risks from Heat-Damaged DNA in Food"

Second Round of Reviewer Comments

Reviewer: 1

Comments to the Author

This research is of significant importance to the field as it sheds light on the potential health risks associated with consuming foods prepared at high temperatures. It highlights the need for further investigation into the potential health impacts of food preparation methods and it will be interesting to see the effects of long-term exposure to heat-damaged DNA in future studies.

Reviewer: 3

Comments to the Author

This paper has been returned to me to review. I see a significant improvement in the revised manuscript and appreciate the careful effort the authors have made. This is rewarding to see, and I think it bodes well that we are closing in on the end of the paper. However, I must point out, as it is an (unfortunate) part of the reviewer's job to raise critical questions and to try to give a reality check, that there are several lingering concerns that were not adequately addressed by the authors in their responses. I hope the authors address these questions in the spirit of trying to make a better paper and to circumvent having such issues appear post publication.

The main issue concerns with Q2 where the following two sub-questions were raised:

A) are the modest fold changes (1.2-1.7x) seen in this current paper from BER-activity-reporter able to be responsibly considered as a functional impact?

B) because many factors/types of perturbations to the cell could alter BER activity, the authors should perform KD/KO of relevant endogenous BER-enzyme(s), since if the fold-changes they observed are indeed BER-dependent, these modest changes are expected to be ablated in KD/KO compared to control lines (and related to this, I had also mentioned general responsibility of 2-3 sh/siRNA-deployment to minimize off-target conclusions)

There was no answer to question 2(A). Perhaps the authors simply expected me to go andread their 2022 ACIEE paper regarding the mitochondrial-DNA BER-activity reporter (UBER)

I did read this cited paper with great interest, and came to learn that the results therein specifically on the KO of two most relevant genes, are far from clear or as predicted, and it is in fact not so genuine to claim that these KO data actually serve to validate this mitochondrial-BER-activity reporter system.

Nonetheless, if the authors already have the KO lines and believe that the reporter system with modest changes is (1) overall robust; and (2) that mtDNA-BER-activity reporter does indeed serve to represent the global/nuclear-DNA-BER activity, and indeed can be reliably used to reflect the damaged nucleotide incorporation into the broader genome, as it's required for the present paper), then they can easily perform these functional checks and balance by comparing their measurements between KO lines and KO-control cells, no?

-Furthermore, the authors responded to Q2B, stating that they had undertaken other further downstream assays. However, as mentioned previously, all of these downstream responses (as well as upstream BER activity changes), can be triggered by multiple causes, and none of these directly report on the point that their damaged nucleotides of interest under the conditions used, are being incorporated into host cell DNA as claimed, especially without the KD/KO functional validations.

-Taken altogether, both the revised and original manuscripts' claims regarding damaged nucleotide incorporation into host DNA following treatment conditions, remain unsubstantiated

Other:

-Fig S8 needs some kind of positive control outcome in parallel, e.g., mitomycin c., as all data / images are showing null signal..

Author's Response to Peer Review Comments:

STANFORD UNIVERSITY, Stanford, California 94305

Eric T. Kool
kool@stanford.edu
George and Hilda Daubert Professor

Tel 650/724-4741
Fax 650/725-0259

Prof. Editor
Senior Editor
ACS Central Science

May 11, 2023

Dear Dr. Editor,

With this letter are submitting our re-revised manuscript entitled "Possible Genetic Risks from Heat-Damaged DNA in Food". In the recent review of the revised paper, the first reviewer recommended for acceptance without change, and the second reviewer also found the manuscript improved but still had questions about the mechanisms involved. In response, we have added further cautions to the manuscript and have revised a figure for clarity. Below is a detailed list of reviewer comments and our responses:

Reviewer #1

Recommendation: Publish in ACS Central Science without change.

Comments:
This research is of significant importance to the field as it sheds light on the potential health risks associated with consuming foods prepared at high temperatures. It highlights the need for further investigation into the potential health impacts of food preparation methods and it will be interesting to see the effects of long-term exposure to heat-damaged DNA in future studies.

We thank the reviewer.

Reviewer #3

Recommendation: Reconsider after major revisions noted.

Comments:
This paper has been returned to me to review. I see a significant improvement in the revised manuscript and appreciate the careful effort the authors have made. This is rewarding to see, and I think it bodes well that we are closing in on the end of the paper. However, I must point out, as it is an (unfortunate) part of the reviewer's job to raise critical questions and to try to give a reality check, that there are several lingering concerns that were not adequately addressed by the authors in their responses. I hope the authors address these questions in the spirit of trying to make a better paper and to circumvent having such issues appear post publication.

We appreciate the reviewer's recognition of our efforts, which involved substantial new work in multiple labs.

The main issue concerns with Q2 where the following two sub-questions were raised:

A) are the modest fold changes (1.2-1.7x) seen in this current paper from BER-activity-reporter able to be responsibly considered as a functional impact?

Yes, we do believe that these statistically significant increases point to a functional impact. We see clearly elevated DNA repair with exposure to these damaged nucleosides. The subsequent data are fully consistent with this, as we see statistically significant downstream effects that are known to result from elevated BER: elevated double-strand breaks (DSB) in cells and animals, and observable chromosomal damage and mutations.

B) because many factors/types of perturbations to the cell could alter BER activity, the authors should perform KD/KO of relevant endogenous BER-enzyme(s), since if the fold-changes they observed are indeed BER-dependent, these modest changes are expected to be ablated in KD/KO compared to control lines (and related to this, I had also mentioned general responsibility of 2-3 sh/siRNA-deployment to

minimize off-target conclusions). There was no answer to question 2(A). Perhaps the authors simply expected me to go and read their 2022 ACIEE paper regarding the mitochondrial-DNA BER-activity reporter (UBER). I did read this cited paper with great interest, and came to learn that the results therein specifically on the KO of two most relevant genes, are far from clear or as predicted, and it is in fact not so genuine to claim that these KO data actually serve to validate this mitochondrial-BER-activity reporter system.

Nonetheless, if the authors already have the KO lines and believe that the reporter system with modest changes is (1) overall robust; and (2) that mtDNA-BER-activity reporter does indeed serve to represent the global/nuclear-DNA-BER activity, and indeed can be reliably used to reflect the damaged nucleotide incorporation into the broader genome, as it's required for the present paper), then they can easily perform these functional checks and balance by comparing their measurements between KO lines and KO-control cells, no? Furthermore, the authors responded to Q2B, stating that they had undertaken other further downstream assays. However, as mentioned previously, all of these downstream responses (as well as upstream BER activity changes), can be triggered by multiple causes, and none of these directly report on the point that their damaged nucleotides of interest under the conditions used, are being incorporated into host cell DNA as claimed, especially without the KD/KO functional validations. Taken altogether, both the revised and original manuscripts' claims regarding damaged nucleotide incorporation into host DNA following treatment conditions, remain unsubstantiated

We respectfully point out that the cellular salvage of damaged nucleotides is presented here as a **hypothesis** (see paragraph 3 and also the abstract), and although it is supported by multiple lines of evidence, we have included many cautions in the paper regarding the mechanism. Note that the title of the paper is not about salvage, but rather about genetic risks upon exposure to these nucleosides that occur in cooked foods, which we believe has been shown convincingly in cells and in mice.

However, the reviewer is correct, that mechanistically there is a slim chance that some overlooked mechanism leads to the genomic damage upon exposure to these nucleosides. To address this, **we have added an additional paragraph which also describes this caution** to the discussion section, below. If such a new mechanism were later identified, it would not invalidate our paper, which makes multiple entirely new and important observations: namely, that cooking damages DNA in food, and that exposure of cells and animals to damaged DNA components found in cooked food causes genetic damage. **This will remain true regardless of the mechanism**.

Respectfully, we cannot agree that our hypothesis of possible salvage of damaged nucleosides and their DNA incorporation remains "unsubstantiated". The UBER probe has been used in BER signaling in three peer-reviewed studies. In our previous published work, we have shown that UBER signal changes depend on BER activity, which has been modulated by repair enzyme knock-out models, multiple inhibitors, DNA damaging agents, and cell cycle analysis. In the current work, we have shown multiple lines of evidence (several of them not dependent on UBER):

(i) addition of the damaged nucleosides enhances UBER signals in multiple cell lines;
(ii) treatment with a dUTPase inhibitor, which is known to accelerate the misincorporation of dU analogues into DNA, further enhances UBER signals upon the addition of dU;
(iii) exposure to the damaged nucleosides resulted in adverse effects related to genomic DNA damage, including mutations and chromosomal breaks;
(iv) treatment with the damaged nucleosides led to increased DSBs measured directly, which is difficult to explain without enhanced BER activity and also points to the DNA location of the damage;
(v) feeding the deaminated nucleoside (dU) to rodents resulted in the accumulation of the corresponding damage in gDNA of intestinal tissue, thus **directly showing salvage of the most common form of damage found after cooking**.

Finally, it should be noted that salvage of modified nucleosides should not be highly controversial, as several modified nucleosides (e.g. BrdU, EdU) are employed widely for labeling genomic DNA in live cells.

We agree that this new hypothesis requires further mechanistic studies in the future for better understanding of the pathway, but we believe that selecting BER enzymes responsible for each damaged nucleoside (some of which are unknown) and testing whether or not this further enhances the BER activity is beyond the scope of this initial work.

Below is the new paragraph added to the Discussion:

Clearly, this initial study is very early, and establishing this hypothesized connection firmly will require more follow-up studies in toxicology. In addition, although our mechanistic hypothesis of salvage of damaged nucleosides is supported by several lines of evidence here (particularly for dU), we cannot yet rule out some unforeseen indirect mechanism whereby exposure to the damaged monomers seen in food elevates cellular DNA damage and subsequent repair responses.

Other:

-Fig S8 needs some kind of positive control outcome in parallel, e.g., mitomycin c., as all data / images are showing null signal.

The positive outcomes of the experiment are shown in Fig. S9. For Fig. S8, we understand the impression that there is nothing in the images, but please see the magnified images attached below; the apparent complete lack of signal is in fact just a very low signal. (below is the image from S8 (dC-fed) without any brightness adjustment; at right is the same image with increased brightness). We can still observe the DSB foci from gamma-H2AX but it is relatively darker compared to the ones from dU-fed mice.

We have now added magnified and enhanced images (labeled as such) to Figs. S8 and S9 to make this clear to readers.

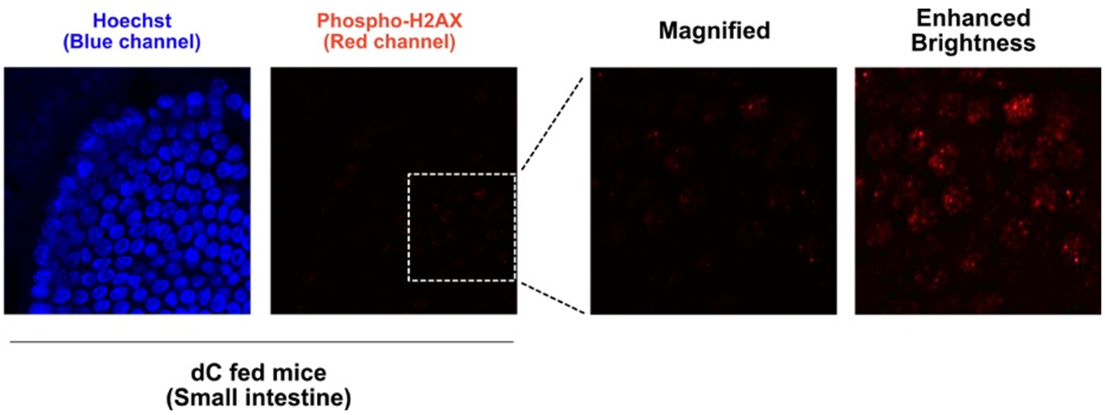

We hope that you now find the manuscript acceptable for publication.

Sincerely,

Eric T. Kool
Department of Chemistry
Stanford University
